# Whole transcriptome characterization of the effects of dehydration and rehydration on *Cladonia rangiferina*, the grey reindeer lichen

**DOI:** 10.1186/1471-2164-14-870

**Published:** 2013-12-10

**Authors:** Sini Junttila, Asta Laiho, Attila Gyenesei, Stephen Rudd

**Affiliations:** 1 Turku Centre for Biotechnology, University of Turku and Åbo Akademi University, Tykistökatu, Turku, Finland; 2 The Finnish Microarray and Sequencing Centre, Turku Centre for Biotechnology, Tykistökatu, Turku, Finland

**Keywords:** Lichen, Desiccation-tolerance, Gene expression, Microarray

## Abstract

**Background:**

Lichens are symbiotic organisms with a fungal and an algal or a cyanobacterial partner. Lichens inhabit some of the harshest climates on earth and most lichen species are desiccation-tolerant. Lichen desiccation-tolerance has been studied at the biochemical level and through proteomics, but the underlying molecular genetic mechanisms remain largely unexplored. The objective of our study was to examine the effects of dehydration and rehydration on the gene expression of *Cladonia rangiferina*.

**Results:**

Samples of *C. rangiferina* were collected at several time points during both the dehydration and rehydration process and the gene expression intensities were measured using a custom DNA microarray. Several genes, which were differentially expressed in one or more time points, were identified. The microarray results were validated using qRT-PCR analysis. Enrichment analysis of differentially expressed transcripts was also performed to identify the Gene Ontology terms most associated with the rehydration and dehydration process.

**Conclusions:**

Our data identify differential expression patterns for hundreds of genes that are modulated during dehydration and rehydration in *Cladonia rangiferina*. These dehydration and rehydration events clearly differ from each other at the molecular level and the largest changes to gene expression are observed within minutes following rehydration. Distinct changes are observed during the earliest stage of rehydration and the mechanisms not appear to be shared with the later stages of wetting or with drying. Several of the most differentially expressed genes are similar to genes identified in previous studies that have investigated the molecular mechanisms of other desiccation-tolerant organisms. We present here the first microarray experiment for any lichen species and have for the first time studied the genetic mechanisms behind lichen desiccation-tolerance at the whole transcriptome level.

## Background

Lichens are symbiotic organisms, which are composed of a fungal (mycobiont) and an algal or a cyanobacterial (photobiont) partner [1]. The fungus is the dominant partner in this symbiosis and provides the algae an optimised space for photosynthesis within the lichen thallus. About 8% of the terrestrial ecosystems are lichen-dominated [2] and lichens live in some of the harshest climates on earth. Lichens are poikilohydric organisms and are unable to actively control the water levels within their thallus. Lichens experience frequent drying and wetting cycles, and they can survive long periods of desiccation in an anhydrobiotic state e.g. when growing on exposed rocks, tombstones and trees. Lichens can also tolerate this desiccation better than either of the isolated symbiotic partners alone [3]. Once lichen is rehydrated, photosynthesis resumes rapidly, typically within minutes [4].

The desiccation tolerance and rapid re-establishment of photosynthesis have been studied at the biochemical level and through proteomics [5-11], but the molecular genetics and functional mechanisms behind these lichen-specific traits remain largely unexplored. The protection against reactive oxygen species (ROS) during desiccation and the subsequent restoration of antioxidant pools during rehydration have been previously investigated using detailed enzymatic assays [12]. Revealing the genetic mechanisms used by lichens to survive long periods of drought could have applications for crop plant development.

*Cladonia rangiferina* (L.) Weber ex F.H.Wigg, the grey reindeer lichen, is a fruticose lichen of the northern European and Arctic regions. It consists of a fungal partner (*C. rangiferina*) and an algal partner (*Asterochloris* sp.), and has been used as our model organism because of its abundance in southern Finland. Our previous investigation of lichen expressed sequence tags [13] identified a number of contig consensus sequences that were annotated using Gene Ontology. These analyses identified candidate actors within the anhydrobiosis systems and established the most basic of genomic foundations required for further molecular genetic analysis of the grey reindeer lichen.

Gene expression studies have previously been used to address the molecular interactions and mechanisms that underlie the broadest range of biological processes that include drought resistance [14] and tolerance [15-17] in addition to the characterization of the molecular interface between other candidate mutualisms [18] and controlled parasitisms. Gene expression profiling may be performed by targeted approaches such as qPCR or *in situ* hybridization or may be performed using more comprehensive genome scale approaches that include the DNA microarray [19] or RNA-Seq [20,21]. A few research studies have been performed that investigate lichen gene expression. Expression has been studied using the more targeted methods of *in situ* hybridization [22,23] and qPCR [24,25]. However, no large or genome scale approaches to study lichen gene expression have yet been published.

We have used the DNA sequence data from our previous investigation of the lichen transcriptome [13] to design a custom DNA microarray for *C. rangiferina* (including probes from both the *Asterochloris* and *Cladonia* partners) in order to identify the transcripts that are expressed in the lichen thallus during dehydration and rehydration. The earlier transcriptome sequences were prepared to sample the gene space and the normalized cDNA libraries were not appropriate for quantitative studies. The aim of this study was to identify the genes most differentially expressed during the rehydration and drying processes and also to establish a more integrative view of the molecular players that contribute to the processes required for lichen desiccation tolerance and the rapid re-establishment of photosynthesis through functional annotation.

## Results

### Sample preparation

**Figure 1:**
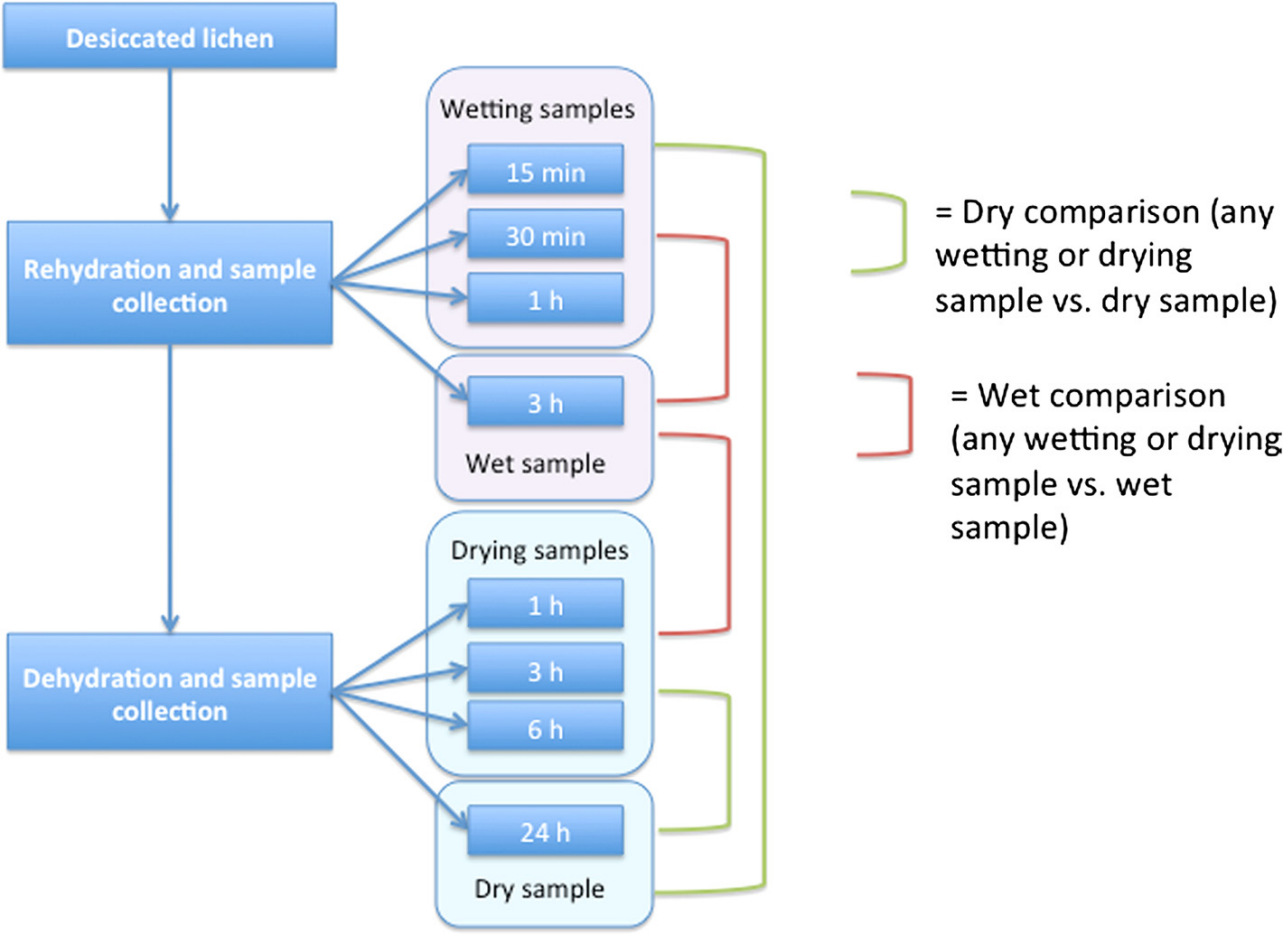
**The experimental procedure and sample set up.** Flowchart illustrating sample set up, the naming of the samples and the different comparisons between the sample groups.

Lichen tissues collected from wild were subjected to a rehydration and desiccation regime. Thallus tissue was sampled at 15 minutes, 30 minutes, 1 hour and 3 hours following rehydration and 1 hour, 3 hours, 6 hours and 24 hours after the commencement of drying. The sample that had been wetting for three hours was considered the wet sample and the sample that had been drying for 24 hours was considered the dry sample. The relative water content (RWC) of the samples was measured during the sample collection. The RWC of the samples during wetting was 13% at 15 minutes, 30.9% at 30 minutes, 62.1% at one hour and 100% at three hours. The RWC of the samples during drying was 45.3% at one hour, 5.7% at three hours, 0% at six hours and 0% at 24 hours. The experimental design is illustrated in Figure 1 and the sample groups and the abbreviations used in this study are summarized in Table 1.

**Table 1:** The sample groups and their abbreviations used in the text

**Sample group name**	**Condition**
W15m	Wetted for 15 min
W30m	Wetted for 30 min
W1h	Wetted for 1 h
Wet	Wetted for 3 hrs
D1h	Air-dried for 1 h
D3h	Air-dried for 3 hrs
D6h	Air-dried for 6 hrs
Dry	Air-dried for 24 hrs

### Lichen oligonucleotide microarray design

Clustered and assembled lichen unigene sequences from deep sequencing and Sanger sequencing data were used to design oligonucleotide probes for the manufacture of a custom DNA microarray. Two oligonucleotide probes were designed for each of the long sequences (arbitrarily defined as ≥ 450 nt) in order to enable the detection of potentially different isoforms of the transcripts. For the sequences shorter than 450 nt (66.1%) a single oligonucleotide probe was designed.

### Data quality control

The distribution of the probe signal intensities from the microarrays was investigated for equivalence through review of box plots (data not shown). Principal component analysis (PCA) of the samples was performed to evaluate group clustering and candidate outlying samples (Additional file 1). The data appeared to be of satisfactory quality and no replicates were removed from the analysis. Of the 41,000 probes designed, an average log2 signal intensity of over 1 was observed for 29,076 of the transcripts in one or more experimental conditions. This shows that the design includes a significant number of genes that are expressed.

The quality of the samples and the sample relations were further investigated using correlation analysis and clustering using Pearson’s metrics (Additional file 1). The between sample correlation values varied between 0.775 and 0.994, whereas the within sample group correlation values were between 0.943 and 0.988 (Additional file 1). This illustrates a high reproducibility between the biological replicates.

### qRT-PCR validation

The transcript identified as *cr_lrc491* was chosen as the endogenous control in the qRT-PCR analysis because it demonstrated similar signal-intensity values across all of the samples, and demonstrated suitability as a control gene. Five other transcripts selected for the qRT-PCR validation (*cr_DMini_2305E01, cr_lrc5, cr_c3825, cr_c4168 and cr_DMini_673H04*) also had steady intensity values across the samples. The transcripts *cr_lrc323, cr_c10766, cr_c15269* and *cr_lrc282* had different expression levels between the samples. Three of the designed assays failed in the qRT-PCR validations. Transcript *cr_c18326* had low intensity levels in all samples and thus probably failed. The amplicon designed in the assay for *cr_DMini_673H04* was 110 bp in length whereas for the other assays the amplicons were 60–70 bp. This could be the reason for the assays failure. The third failed assay was *cr_lrc282*.

**Figure 2:**
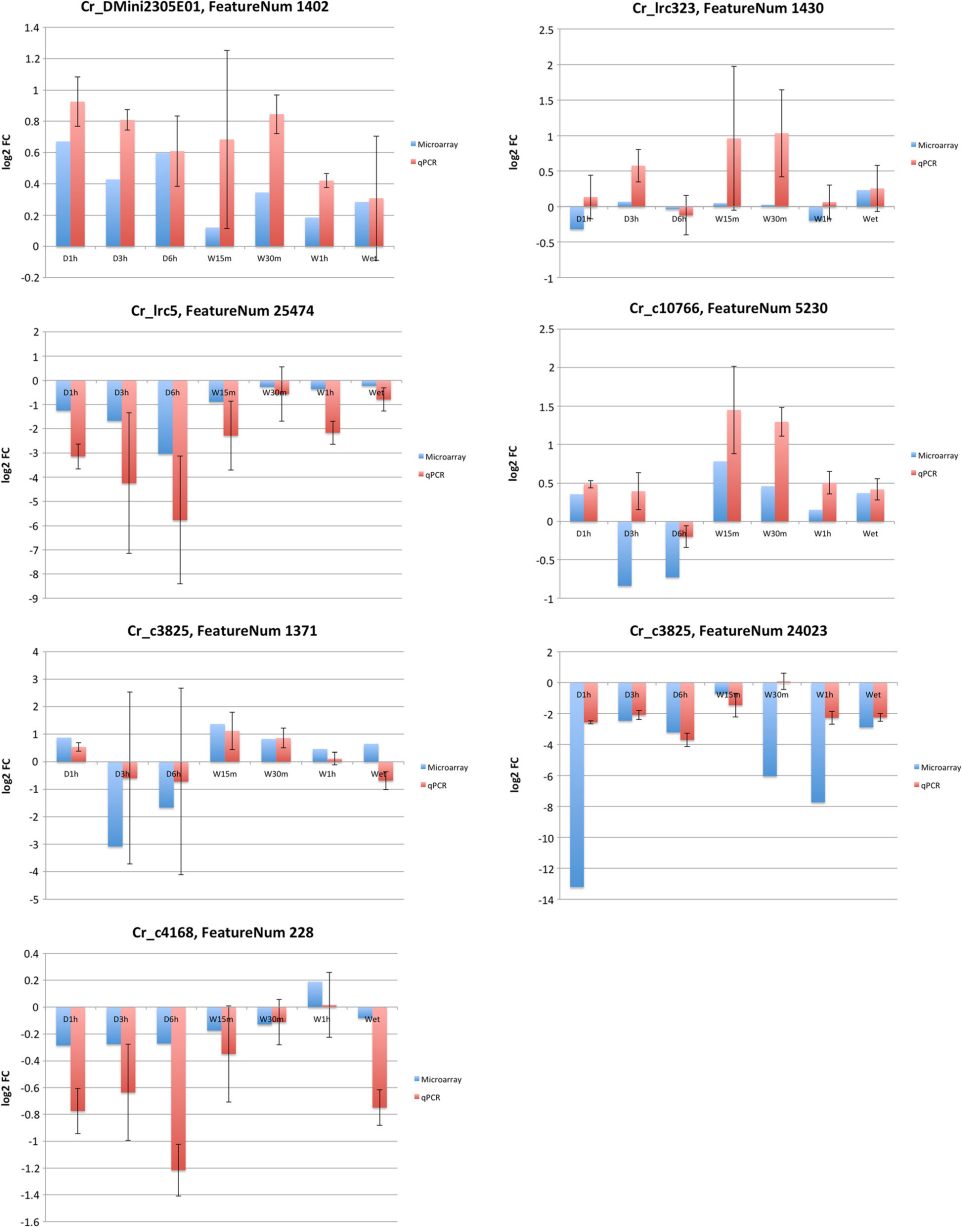
**The validation of the microarray results using quantitative real-time RT-PCR.** The qRT-PCR values are the mean of four replicate measurements of each of the three samples in the sample group. The microarray values are obtained from linear modelling results, which are calculated from the average expression intensities of the three replicates in each sample group, and therefore variance values are not available. Both microarray and qRT-PCR results are compared to the Dry sample group.

The microarray and qRT-PCR results corresponded well (Figure 2) with 91.1% of the measurements giving similar results with both methods. The relative quantitation values of some transcripts varied significantly between the samples in some sample groups in the qRT-PCR analysis (Additional file 2).

### Annotation

The microarray design includes 40,924 oligonucleotide probes for analysis of gene expression in lichen samples. Since *C. rangiferina* is a symbiotic organism it is composed of both fungal and algal partners and the probes on the DNA microarray may represent either fungal or algal genes. The genome of origin for the lichen EST sequences used in the microarray probe design was predicted using Eclat [26]. 29.6% of the EST sequences used in the microarray probe design are predicted to be of algal origin while the remaining 70.4% are predicted to be fungal sequences. The classification is included in the annotation of the full differentially expressed gene lists available as Additional files 3, 4, 5, 6, 7, 8, 9, 10, 11, 12, 13, 14, 15.

Since no lichen reference genome has yet been published, an annotation of the transcript sequences was performed using a BLASTX homology-based approach. Annotation could not be assigned to 15,816 probes (38.6%) because their target sequences did not demonstrate a BLAST match exceeding the required statistical threshold of 1e-3. 13,852 (33.9%) transcript sequences corresponded to NCBI non-redundant database entries annotated as “conserved hypothetical proteins”, “hypothetical proteins” or “predicted proteins”. 27.5% of transcript sequences could be assigned to an unambiguous and natively annotated reference protein.

### Differentially expressed transcripts

The thresholds for assigning differentially expressed (DE) transcripts from either the mycobiont and photobiont were selected from the plotted volcano plots and heat maps (Additional files 16 and 17). The numbers of DE transcripts in the different comparisons are shown in Table 2. The full DE transcript lists for each comparison are available as Additional files 3, 4, 5, 6, 7, 8, 9, 10, 11, 12, 13, 14, 15.

**Table 2:** Filtering parameters for all comparisons and the amount of differentially expressed transcripts

**Comparison**	**FC**	**logFC**	**P type**	**P**	**Total**	**Up**	**Down**
W15m vs. dry	32	5	FDR	0.001	1108	531	577
W15m vs. wet	32	5	FDR	0.001	931	348	583
W30m vs. dry	32	5	FDR	0.001	572	442	130
W30m vs. wet	8	3	FDR	0.01	560	402	158
W1h vs. dry	32	5	FDR	0.001	595	398	197
W1h vs. wet	8	3	P value	0.001	162	80	82
Wet vs. dry	16	4	FDR	0.001	675	589	86
D1h vs. dry	16	4	FDR	0.001	980	599	381
D1h vs. wet	16	4	FDR	0.001	919	393	526
D3h vs. dry	32	5	FDR	0.001	645	430	215
D3h vs. wet	32	5	FDR	0.001	614	266	348
D6h vs. dry	32	5	FDR	0.001	663	462	201
D6h vs. wet	32	5	FDR	0.001	624	317	307

FC = fold change, logFC = 2-logarithmic fold change, P = p value, FDR = false discovery rate, Total = total amount of differently expressed transcripts, Up = amount of up-regulated transcripts, Down = amount of down-regulated transcripts.

The number of the differentially expressed transcripts differs between the comparisons; W1h vs. Wet shows only 162 DE transcripts while W15m vs. Dry comparison yielded 1,108 DE transcripts. Also the ratio of up- and down-regulated transcripts varies from comparison to comparison. The W1h vs. Wet comparison reveals an even distribution where approximately half of the DE transcripts are up-regulated. Wet vs. Dry comparison suggests 589 up-regulated transcripts and only 86 down-regulated transcripts.

The DE transcripts from each comparison were compared to one another to find out the number of transcripts that the comparisons have in common (Additional file 18). Uniformity of expressed transcripts is observed across both Dry and Wet comparisons, and across the wetting and drying sample groups with the exception of group W15m.

The most differentially expressed transcripts do not have clear sequence orthologues or show sequence similarity to only hypothetical proteins. An analysis of the DE transcripts was performed using only the top DE transcripts with annotation derived from primary annotation sources rather than passaged secondary annotations (Table 3). The most common up-regulated genes were CaaX farnesyltransferase alpha subunit, U5 snRNP component, nuclear pore complex subunit Nup192, Swr1p complex component and cytochrome P450 family protein. The most common down-regulated genes on the other hand were heat shock protein HSP98, ion channel, nitrite reductase and siroheme synthase.

**Table 3:** The top five annotated genes for each comparison

**Wetting sample groups vs. wet**								
**Annotation**	Up W15m vs. wet	Down W15m vs. wet		Up W30m vs. wet	Down W30m vs. wet	Up W1h vs. wet		Down W1h vs. wet
	Short-chain dehydrogenase, putative, F	Aspartic-type endopeptidase, putative, F		Short-chain dehydrogenase, putative, F	Nitrite reductase, F	CaaX farnesyltransferase alpha subunit, F		Nitrite reductase, F
	Mitochondrial molecular chaperone, F	Enoyl-acyl-carrier-proteinreductase 1, F		CaaX farnesyltransferase alpha subunit, F	30 kDa heat shock protein, F	Beta-glucosidase, putative, F		30 kDa heat shock protein, F
	Alcohol dehydrogenase, F	NEDD8 conjugating enzyme, F		Mitochondrial molecular chaperone, F	Ion channel, F	Non-classical export protein Nce102, putative, F		Siroheme synthase, putative, F
	PaaI thioesterase family protein, putative, F	DNA-directed RNA polymerase II subunit, F		Beta-glucosidase, putative, F	Hsp98/Hsp104/ClpA, putative, F	40S ribosomal protein, A		Ion channel, F
	Membrane-spanning ATPase, F	Eukaryotic translation initiation factor 3, F		Alcohol dehydrogenase, F	Hsp98, F	Vesicle coat complex COPII, subunit Sec24 family protein, F		DNA binding protein SART-1, F
**Wetting sample groups vs. dry**								
**Annotation**	Up W15m vs. dry	Down W15m vs. dry	Up W30m vs. dry	Down W30m vs. dry	Up W1h vs. Dry	Down W1h vs. dry	Up Wet vs. dry	Down Wet vs. dry
	Short-chain dehydrogenase, putative, F	Enoyl-acyl-carrier-proteinreductase 1, F	Nuclear pore complex subunit Nup192, putative, F	Ion channel, F	Nuclear pore, complex Nup192, putative, F	Ion channel, F	DNA-directed RNA polymerase, F	Ion channel, F
	Nuclear pore complex subunit Nup192, putative, F	Aspartic-type endopeptidase, putative, F	Riboflavin aldehyde-forming enzyme, F	Nitrite reductase, F	Swr1p complex component, F	Nitrite reductase, F	Cytochrome P450 family protein, F	Imidazole glycerol phosphate synthase, A
	Mitochondrial molecular chaperone, F	NEDD8 conjugating enzyme, F	DNA-directed RNA polymerase, F	30 kDa heat shock protein, F	Riboflavin aldehyde-forming enzyme, F	Siroheme synthase, putative, F	Karyopherin, F	26S protease regulatory subunit, A
	PaaI thioesterase family protein, putative, F	Eukaryotic translation initiation factor 3, F	Cytochrome P450 family protein, F	Siroheme synthase, putative, F	Cytochrome P450 family protein, F	30 kDa heat shock protein, F	Swr1p complex component, F	40S ribosomal protein, A
	Alcohol dehydrogenase, F	DNA-directed RNA polymerase II subunit, F	Swr1p complex component, F	Copia-type polyprotein, F	DDHD domain protein, F	Imidazole glycerol phosphate synthase, A	Riboflavin aldehyde-forming enzyme, F	Molecular chaperone, F
**Drying sample groups vs. dry**								
**Annotation**	Up D1h vs. dry	Down D1h vs. dry		Up D3h vs. dry		Down D3h vs. dry	Up D6h vs. dry	Down D6h vs. dry
	Nuclear pore complex subunit Nup192, putative, F	Ion channel, F		Nuclear pore complex subunit Nup192, putative, F		Ion channel, F	Nuclear pore complex subunit Nup192, putative, F	Ion channel, F
	UDP-glucose 4-epimerase, F	Nitrite reductase, F		T-complex protein 1, gamma subunit, F		Amino acid permease, putative, F	Phospholipase, F	Siroheme synthase, putative, F
	Karyopherin, F	Siroheme synthase, putative, F		Phospholipase, F		Siroheme synthase, putative, F	DDHD domain protein, F	Siroheme synthase, F
	Swr1p complex component, F	Siroheme synthase, F		Swr1p complex component, F		Translation initiation regulator, putative, F	T-complex protein 1, gamma subunit, F	Amino acid permease, putative, F
	Cytochrome P450 family protein, F	Siroheme synthase, N-terminal domain containing protein, F		DDHD domain protein, F		Cation-transporting ATPase, F	Cytochrome P450 family protein, F	Cation-transporting ATPase, F
**Drying sample groups vs. wet**								
**Annotation**	Up D1h vs. Wet	Down D1h vs. Wet		Up D3h vs. Wet		Down D3h vs. Wet	Up D6h vs. Wet	Down D6h vs. Wet
	Dynamin GTPase, A	Nitrite reductase, F		Phospholipase, F		Cation-transporting ATPase, F	Phospholipase, F	Cation-transporting ATPase, F
	Major royal jelly protein, F	Siroheme synthase, N-terminal domain containing protein, F		Mitochondrial carrier protein, putative, F		Translation initiation regulator, putative, F	Mitochondrial carrier protein, putative, F	Hsp98, F
	CaaX farnesyltransferase alpha subunit, F	Hsp98, F		Nitrogen metabolite repression regulator, F		Hsp98, F	Benzoate 4-monooxygenase cytochrome P450, F	Ferric reductase NAD binding domain containing protein, A
	U5 snRNP component, putative, F	Translation initiation factor IF-2, F		U5 snRNP component, putative, F		Ferric reductase NAD binding domain containing protein, A	MFS monosaccharide transporter, putative, F	bZIP transcription factor HacA, F
	Vesicle coat complex COPII, subunit Sec24 family protein, putative, F	Ion channel, F		MFS monosaccharide transporter, putative, F		Polyketide synthase, putative, F	U5 snRNP component, putative, F	Ion channel, F

Up = up-regulated genes, down = down-regulated genes, A = algal sequence, F = fungal sequence.

The W15m sample group had only one gene in common with the other comparison groups; the nuclear pore complex subunit Nup192 in up-regulated W15m vs. Dry comparison. However, four of the five top annotated up-regulated DE genes and all top five annotated down-regulated DE genes were the same in W15m vs. Wet and W15m vs. Dry comparisons.

### Hierarchical clustering of the expression values of the annotated transcripts

**Figure 3:**
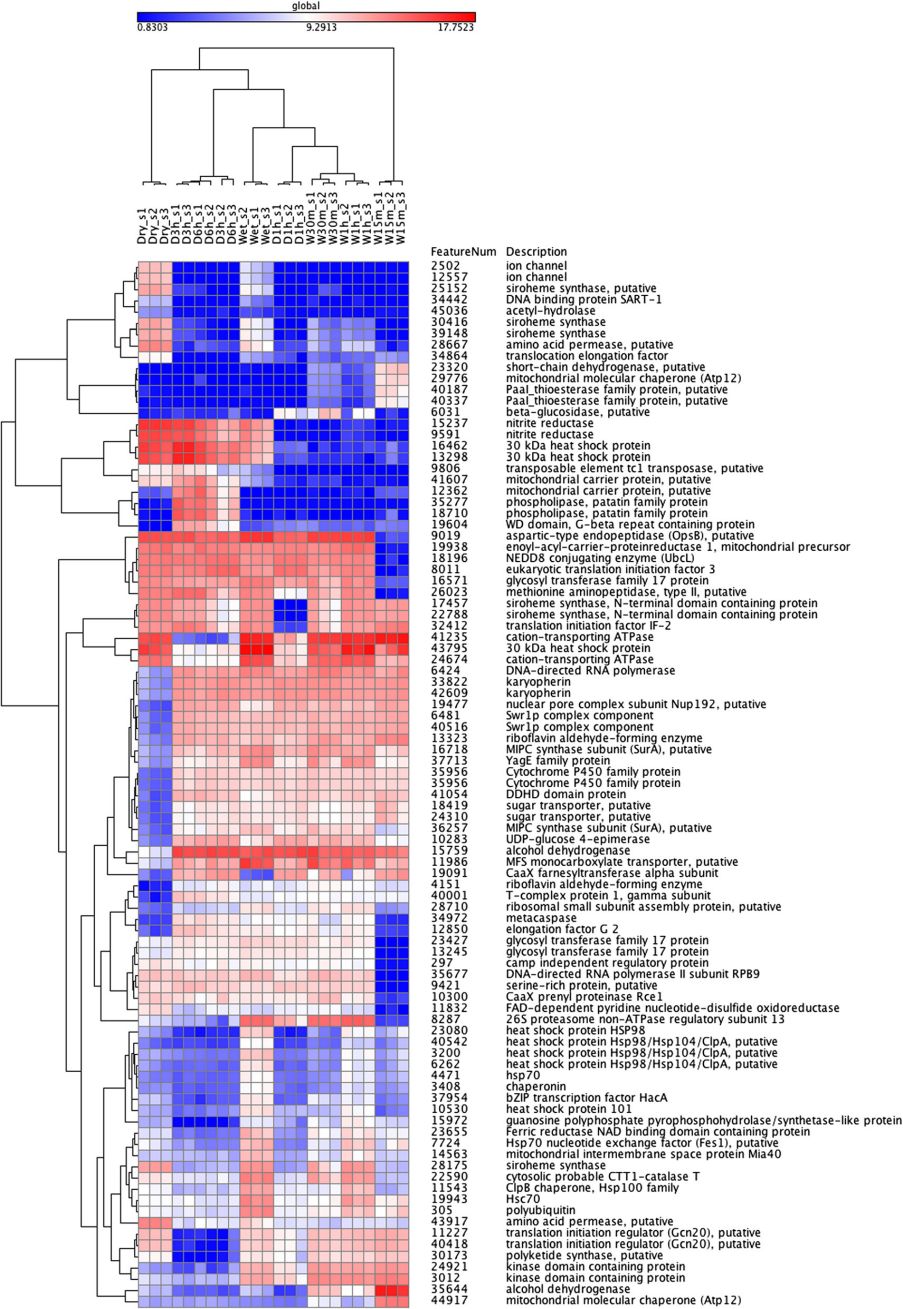
**Hierarchical clustering of the expression values.** Hierarchical clustering of the top 20 well-annotated genes from each comparison. Blue colour represents low expression values, red colour high expression values.

The expression values of the genes with primary annotations were clustered across all samples to investigate trends in the differential expression of genes across the sample groups. The clustering of the top 20 well-annotated DE genes is shown in Figure 3, for the full clustering heat map see Additional file 19. Similar to the clustering of the individual sample groups (Additional file 1), the W15m samples differ significantly from the other samples in this heat map. The drying and wetting samples form the two main clusters.

### Functional analysis of the DE gene lists

Functional annotation was performed using Gene Ontology (GO) and Kyoto Encyclopedia of Genes and Genomes (KEGG) databases. For the functional analysis, lower threshold values for both fold change (FC > |1.5|) and p-value (p-value < 0.01) were applied in order to include a larger number of transcripts in the input list. With these less stringent thresholds more transcripts were selected and finer changes within GO terms can be observed. The full tables containing the over-represented GO terms for each comparison are presented as Additional file 20. An enrichment analysis of KEGG pathways was also performed, but an insufficient number of genes could be assigned to pathways to enable an interpretable analysis.

## Discussion

Lichens are resilient organisms and can endure desiccation for long periods at a time. Even after a long period of desiccation they can resume photosynthesis rapidly, typically within minutes, upon rehydration. Lichen desiccation tolerance has been studied at the biochemical level [6,7,9], but little is known about the molecular mechanisms behind this interesting phenomenon and ability to survive in harsh environments. We have studied the changes in gene expression of *Cladonia rangiferina* during drying and wetting by designing a custom microarray. The objective of our research was to obtain a better insight into the desiccation tolerance of *C. rangiferina* and identify the genes most differentially expressed during drying and wetting.

The reindeer lichen is a symbiotic organism containing a mycobiont (*C. rangiferina*) and an algal photobiont (*Asterochloris* sp.). The expressed transcripts in the lichen thallus may be derived from either of these genomes. Since we specifically intended to study desiccation- and rehydration-induced gene expression of the whole symbiotic lichen, which has been shown to tolerate desiccation better than either of its isolated partners [3], the microarray design was based on sequences prepared from whole lichen tissue. The genome of origin for these poly-adenylated cDNA-based EST sequences have been classified as fungal or algal sequences on the basis of discriminative models derived using available axenically cultured sequences [13] to obtain an estimate of the ratio of algal and fungal genes in the lichen.

Similarly also the RNA samples, which were hybridized to the microarray, contain RNA molecules from both of the symbionts. Lichens are also known to contain internal bacterial communities [27] and bacterial contamination is a possibility with sample material collected from the wild, but the bacterial RNA molecules possibly present in the samples should not hybridize to lichen based microarray probes.

Samples were collected at different time points during rehydration and desiccation and the RWC was measured from each time point studied. 24 hours was selected as the final sample time-point because beyond this point no changes in lichen tissue weight were observed. The starting lichen material was dried only in room temperature under normal laboratory conditions and therefore the RWC of the starting material can be higher than measured. The D6h and Dry samples could have obtained moisture from air humidity during the drying process thus explaining why we were able to detect transcriptomic effects in these samples although the RWC values were so low.

The correlation values between the samples show variability, but the within group correlation values are high (Additional file 1) indicating heterogeneous sample groups and homogenous replicates within sample groups. The metabolic processes taking place during wetting and desiccation were hypothesized to be large, systemic changes, which is reflected with the lower between sample correlation values and the high number of differentially expressed transcripts despite the strict filtering thresholds (Table 2).

Within the DE transcript lists (Additional files 3, 4, 5, 6, 7, 8, 9, 10, 11, 12, 13, 14, 15), the percentage of unannotated genes is enriched when compared to the whole data. In many of the comparisons the percentage of unannotated DE transcripts is close to 50% and the highest percentage is 66.3% within the Wet vs. Dry comparison. This enrichment of unannotated transcripts within the strictly filtered lists could suggest that these may be lichen-specific or -adapted genes. It would also seem likely that these lichen-specific transcripts would be differentially expressed especially during the early stages of wetting and drying. When using more permissive thresholds for filtering the distribution of unannotated transcripts appears similar to the whole data (results not shown) illustrating that these putative lichen-specific transcripts really are key players in the molecular mechanisms underpinning anhydrobiosis. The most abundantly represented transcripts in the rehydrated *Tortula ruralis*, a desiccation-tolerant bryophyte, also did not have any matches to known sequences [28].

Our results suggest that the molecular responses happening in the beginning of rehydration are different from the later stages of wetting and also from drying. This can be seen at the level of the whole data (Additional file 1), as the difference of the expression profile of the top DE genes in W15m sample (Figure 3) and also as the uniqueness of the top up- and down-regulated genes in the W15m comparisons (Table 3). The enriched up-regulated GO terms in W15m sample suggest that cellular responses involved in molecule transport and localization (Additional file 20) are active in the early stages of rehydration.

The differential expression of some genes, like short chain dehydrogenase and alcohol dehydrogenase (Table 3), and the enrichment of certain GO terms continues past the initial rehydration. Alcohol dehydrogenases take part in detoxification reactions [29], and therefore their up-regulation could reflect the detoxification of ROS and other harmful metabolites accumulated during desiccation. Alcohol dehydrogenase deficient plants have been shown to be more susceptible to osmotic stress in tomato [30] and *Arabidopsis thaliana*[31], and as alcohol dehydrogenase family proteins function in the polyol synthesis pathway, their activity could contribute towards protection against water deficiency [32]. Transcripts are known to accumulate during drying in desiccation-tolerant plants [33] minimizing the time needed to restart growth upon rehydration. Several GO terms involved in molecule transport are enriched in the W15m sample as well as in the W30m sample, and could potentially reflect this activation of pools of transcripts available in the lichen tissue.

As the rehydration continues, biosynthetic and metabolic processes become more active in *C. rangiferina* as suggested by the enriched GO terms in the W30m sample (Additional file 20). These processes are active also in the W1h sample (Additional file 20) and it would seem logical that processes involved in the synthesis of proteins and other molecules are activated in the rehydrated lichen. However, it seems that these biosynthetic and metabolic processes are less active in the Wet sample than in the W30m and W1h samples. The cellular responses that encompass the entire rehydration process are all related to the binding of nucleic acids.

The up-regulation of the heat shock protein HSP98 (Table 3) and the enrichment of response to stress term (Additional file 20) in the Wet sample indicate that already three hours’ full hydration is a source of stress for *C. rangiferina*. Heat shock proteins are induced in various environmental stress responses [34], and it has previously been found that desiccation-tolerant lichens find 48 hours storage in moist mildly stressful [8]. Desiccation-tolerant lichens are in general more adapted to short bursts of hydration with long intervals of desiccation and therefore long periods of full rehydration may potentially be a stressful situation for them.

When the lichen starts to dry, the gene expression profiles are similar to those observed during rehydration (Figure 3). After three to six hours of dehydration several biosynthetic and metabolic processes are still active in the lichen (Additional file 20), but the expression profile has changed (Figure 3). The acyl-transferase activities increase during these later stages of dehydration (Additional file 20) and the up-regulation of U5 snRNP component gene was detected during the whole drying process (Table 3). A U5 snRNP-associated protein is up-regulated by cold stress in *Arabidopsis*[35] and smallRNAs as well as ribosome binding proteins are involved in regulating plant responses to abiotic stresses [36,37].

According to our results, ion channel is up-regulated in the Dry sample (Table 3). Damage to the plasma membrane may be the main cause of death during desiccation [38], and resurrection plants have been shown to utilize cell wall modification to enhance desiccation tolerance [39]. This observed up-regulation of a transcript coding for an ion channel protein could potentially reflect the protection of cellular integrity that is found integral for desiccation tolerance in plants [37].

Our findings suggest that some genes are involved in both the rehydration and dehydration process. Nuclear pore complex subunit Nup192, Swr1p complex component and cytochrome P450 family protein genes are differentially expressed in most drying and wetting samples (Table 3). Nup192 is an evolutionarily conserved nucleoporin with a preferential location at the inner site of the nuclear membrane [40], and nucleoporins have been shown to play critical roles in gene regulation [41]. In plants nucleoporins have been found necessary to survive cold stress [42], and essential for the symbiosis with mycorrhizal fungi [43]. Swr1p is a member of the Swi2/SNF2 family of ATP-dependent chromatin remodelling enzymes [44], and it regulates the deposition of histone at repressed promoters and allows for the rapid activation of transcription in yeast [45]. A gene encoding a SNF2 domain-containing protein was identified as dehydration-upregulated in the resurrection plant *Xerophyta humilis*[32]. Cytochrome P450 proteins on the other hand have been shown to catalyse a wide variety of reactions in plant [46,47] and fungal [48,49] primary and secondary metabolism and the production of secondary metabolites often depends on many species-specific P450s [50]. A cytochrome P450 enzyme transcript has been shown to be significantly accumulated in rapidly dried and subsequently rehydrated *Tortula ruralis*[17].

While we have demonstrated the differential expression of hundreds of genes in the drying and wetting lichen, these changes all occur within hours of profound changes to water content. In field conditions there will likely be finer changes as atmospheric moisture partially rewets the lichen. This study does not address the anhydrobiosis phenomena where tissues can remain viable for months under the dried conditions – this is likely controlled well beyond the 24 hour point to which we characterized the drying process. This study does however provide insight into the molecular activities associated with the beginning dehydration and has identified many apparently novel genes that will be of value in subsequent studies.

## Conclusions

We have studied the changes in gene expression of *Cladonia rangiferina* during rehydration after desiccation and dehydration after full hydration. Our data suggests that these events affect the expression levels of hundreds of genes at a statistically significant level. The wetting and drying events differ from each other at the molecular level, but the largest changes to gene expression are observed minutes after rehydration. These changes are unique to the early stages of rehydration and are not shared with the later stages of wetting or with drying. Due to lack of annotation for lichen sequences, most of the differentially expressed transcripts are currently novel showing no sequence similarity to known genes. These candidate lichen-specific genes may contribute to the molecular mechanisms required by the organism to tolerate long periods of desiccation. Several of the identified genes are similar to genes identified in other studies analysing the desiccation mechanisms of desiccation-tolerant organisms. An analysis of the functional annotation of differentially expressed genes was also performed and it gives an illustrative view of the different biological responses displayed in the lichen thallus during rehydration and desiccation. We have presented here the first microarray experiment for any lichen species and have for the first time studied the genetic mechanisms behind lichen desiccation-tolerance at the whole transcriptome level.

## Methods

### Sample preparation and RNA extraction

Lichen was collected from the island of Kuusisto in Kaarina, Finland on 2nd of May, 2009 and after removal of other plant material and small invertebrates stored at -20°C in desiccated state. Rewetting of the lichen material was performed following the overnight thawing of the frozen samples at room temperature. The lichen tissue was rewetted by spraying with tap water, and placing the material on a wet dishcloth under standard laboratory conditions. Samples for the microarray experiment were collected at 15 minutes, 30 minutes, one hour and three hours after rewetting. After three hours of wetting the lichen tissue was left to dry at room temperature on a dry cloth and samples were collected at one, three, six and 24 hours of drying. The RWC of the samples was measured by weighing the samples. Three replicates of each sample were collected.

The lichen tissue samples were placed in liquid nitrogen immediately following collection and the tissue was finely ground using a mortar and pestle. RNA was extracted with the Spectrum Plant kit (Sigma Aldrich, Germany) according to the manufacturer’s instructions. The concentration and quality of the isolated nucleic acids was measured using the NanoDrop ND-1000 (NanoDrop, USA) and RNAs were stored at -80°C.

The culturing of the axenically grown symbiotic partners and the generation of EST sequences from both fungal and algal partners was performed as previously described [13].

### Microarray design

Our lichen custom microarray was manufactured according to the Agilent 4x44K array format and oligonucleotide probes were designed and optimised using the manufacturer’s eArray tool ( https://earray.chem.agilent.com/earray/). Previously published Roche GS FLX deep sequencing reads and EST sequences obtained by traditional Sanger sequencing [13] were used as substrate for the array design process. The Roche GS FLX reads were assembled with MIRA2 [51] and the Sanger sequences base called using phred [52] and compared against a modified NCBI UniVec database using cross_match to identify vector and polylinker sequence substrings. These lichen transcriptome sequences were split into two groups according to the nucleotide length of the sequence, sequence contigs of less than 450 nt in length were classified as short sequences while sequences longer than or equal to 450 nt in length were classified as long. For the 20,779 short contigs a single 60 nt probe per target sequence was designed while for the 10,676 long sequences two 60 nt probes per target sequence were designed. This resulted in 20,663 probes for the short sequences and 20,071 probes for the long sequences. In addition a replicate probe group of 19 probes was created as recommended by Agilent. These probes were each replicated ten times on the array. Agilent control probes were additionally included into the array design.

### Microarray hybridization

Prior to microarray analysis, the quality of the starting total RNA was validated using 2100 Bioanalyzer (Agilent, USA) capillary electrophoresis instrument. All of the samples had a RNA Integrity Number (RIN) [53] value above 9. The RNA sample labelling and hybridization was performed using the manufacturer’s One-Color Microarray-Based Gene Expression Analysis protocol (Agilent, USA, Version 5.7).

600 ng of total RNA was amplified and labelled with Cy3 using the Quick Amp Labeling kit (Agilent USA). The samples were processed with an exogenous control sample provided through an RNA Spike-in kit (Agilent, USA). 1.65 μg of Cy3-labelled sample was hybridized to the 4x44K custom array in 65°C overnight using the provided Gene Expression Hybridization kit. The arrays were washed using the Gene Expression Wash Pack and Stabilization and Drying solutions also provided in the custom DNA microarray gene expression profiling kit.

The DNA microarrays were scanned using an Agilent Technologies’ Scanner model G2565CA. The expression data was derived from the image files using Agilent’s Feature Extraction software, version 10.5.1.1, using grid 024161_D_F_20090605, protocol GE1_105_Dec08 and QC metric set GE1_QCMT_Dec08. These microarray data have been deposited in NCBI’s Gene Expression Omnibus [54] and are accessible through GEO Series accession number GSE47624 ( http://www.ncbi.nlm.nih.gov/geo/query/acc.cgi?acc=GSE47624).

### Quantitative real-time RT-PCR validations

Eleven transcripts were selected for qRT-PCR validation. The primers were designed using Roche’s Universal Probe Library with the Probe Finder software version 2.45 ( http://qpcr.probefinder.com/organism.jsp). An optimal real-time PCR assay was successfully designed for all of the selected transcripts and the primers were manufactured by Oligomer Ltd (Helsinki, Finland). The primer sequences are provided as Additional file 21.

1 μg of RNA was reverse transcribed into cDNA using Dynazyme reverse transcriptase (Finnzymes, Finland). qRT-PCR reactions were performed for each sample in four replicates using KAPA qPCR Master Mix (Kapa Biosystems, USA) with the following amplification protocol: 10 minutes at 95°C, 40 cycles of 15 s at 95°C and 1 min at 60°C, 10 s at 8°C. The transcript identified as *cr_lrc491* was used as an endogenous control and fold change values compared to the Dry sample were calculated using the RQ Manager v.1.2 software (Life Technologies, USA).

### Data analysis

The oligonucleotide probe identifiers and signal intensities from the scanned microarray image files were determined using software running on the microarray scanner (Feature Extraction, v 10.5.1.1, Agilent). These raw data were analysed using R and Bioconductor [55,56]. The data were quantile normalized to reduce technical noise using Limma package [57]. Each time point during dehydration and rehydration was compared to both the Wet and Dry sample to identify the differentially expressed genes in each of these comparisons. The Limma package, which applies linear modelling with a modified t-test, was used for the statistical testing and the thresholds for the up- and down-regulated genes were chosen individually for each comparison (Table 2, Additional files 17 and 18).

Eclat classification of the lichen EST sequences used for the microarray probe design was performed as previously described [13]. BLASTX [58] was used to compare the lichen sequences to a non-redundant (nr) protein sequence database from the NCBI GenBank database [59] using an arbitrary cut-off of 1e-3.

The Blast2GO [60] tool was used to analyse the BLASTX sequence results. This analysis was used to assign candidate GO [61] and enzyme code (EC) [62] annotations, to perform Interpro scans [63] and to identify candidate protein sequence domains and to map sequences onto KEGG pathways. Each sample group comparison was tested for enrichment of GO terms using the Fisher’s Exact Test method as implemented in Blast2GO. A fold-change threshold of > |1.5| and a p-value threshold of 0.01 was assigned for determination of sensitivity for the Fisher’s Exact Test. A non-two-tailed test with p-value threshold of 0.05 was used to distinguish the over-represented GO terms in each sample group comparison.

The clustering of the transcript expression values and the plotting of their heat maps was performed using the GENE-E tool ( http://www.broadinstitute.org/cancer/software/GENE-E/index.html). Euclidean clustering was selected for the clustering method and average linkage as the linkage method. Logarithmic expression values were used in the input data.

### Availability of supporting data

The data set supporting the results of this article is available in the Gene Expression Omnibus repository, GEO Series accession number GSE47624, http://www.ncbi.nlm.nih.gov/geo/query/acc.cgi?acc=GSE47624.

## Competing interests

The authors declare that they have no competing interests.

## Authors’ contributions

SJ performed the lab experiments and most of the data analyses and drafted the manuscript. AL wrote the R code for the data analysis. SR performed the assembly of the deep sequencing reads. AG helped with the data analysis. All authors participated in the design of the experiments and read and approved the final manuscript.

## Supplementary Material

Additional file 1 Figures used in assessment of data quality in compressed format. Click here for file

Additional file 2 qRT-PCR results. Click here for file

Additional file 3 The full DE transcript list for comparison D1h vs. Dry. Click here for file

Additional file 4 The full DE transcript list for comparison D1h vs. Wet. Click here for file

Additional file 5 The full DE transcript list for comparison D3h vs. Dry. Click here for file

Additional file 6 The full DE transcript list for comparison D3h vs. Wet. Click here for file

Additional file 7 The full DE transcript list for comparison D6h vs. Dry. Click here for file

Additional file 8 The full DE transcript list for comparison D6h vs. Wet. Click here for file

Additional file 9 The full DE transcript list for comparison W15m vs. Dry. Click here for file

Additional file 10 The full DE transcript list for comparison W15m vs. Wet. Click here for file

Additional file 11 The full DE transcript list for comparison W30m vs. Dry. Click here for file

Additional file 12 The full DE transcript list for comparison W30m vs. Wet. Click here for file

Additional file 13 The full DE transcript list for comparison W1h vs. Dry. Click here for file

Additional file 14 The full DE transcript list for comparison W1h vs. Wet. Click here for file

Additional file 15 The full DE transcript list for comparison Wet vs. Dry. Click here for file

Additional file 16 Volcano plots for each comparison in compressed format. Click here for file

Additional file 17 Heat maps for each comparison in compressed format. Click here for file

Additional file 18 The amount common differentially expressed transcripts in the comparisons. Click here for file

Additional file 19 Clustering heat map of the DE genes. Click here for file

Additional file 20 GO term enrichment files in compressed format. Click here for file

Additional file 21 qRT-PCR primer sequences. Click here for file
