# Supplementary figures and images for: Whole transcriptome characterization of the effects of dehydration and rehydration on Cladonia rangiferina, the grey reindeer lichen
Source: BMC Genomics. 2013 Dec 10;14:870. doi: 10.1186/1471-2164-14-870 (PMC3878897; doi:10.1186/1471-2164-14-870)

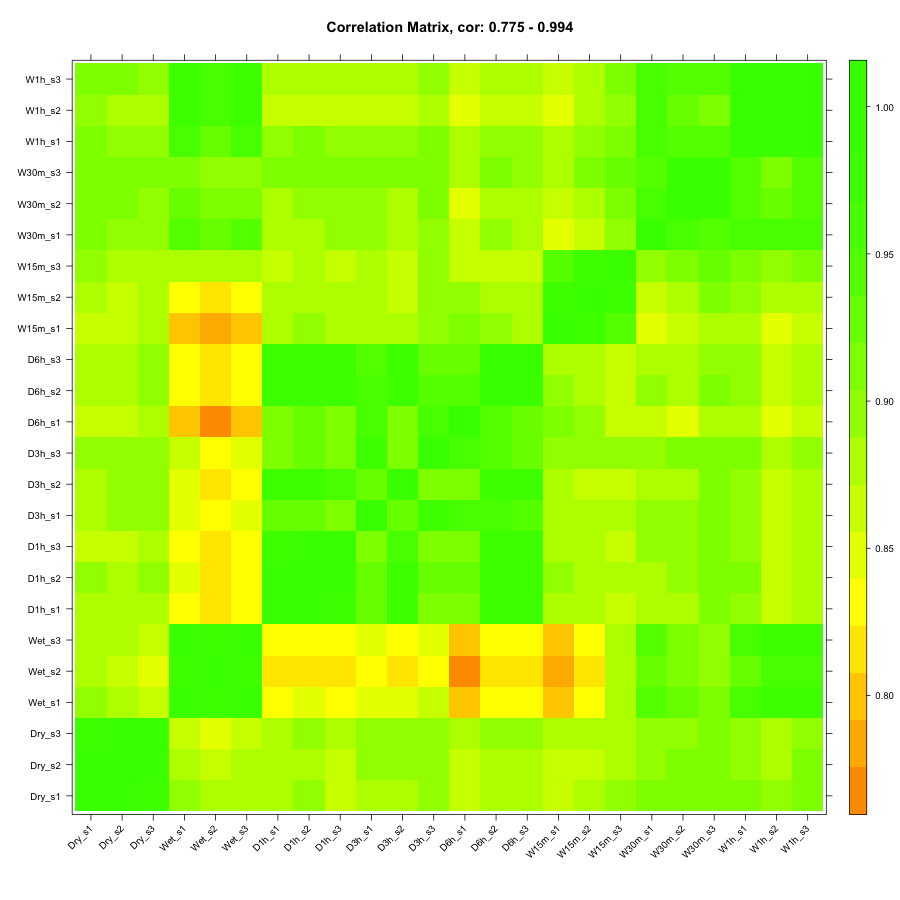

Supplement: Additional file 1 — Figures used in assessment of data quality in compressed format. [file 1471-2164-14-870-S1.zip › DataQualityControl/correlationMatrix.png]

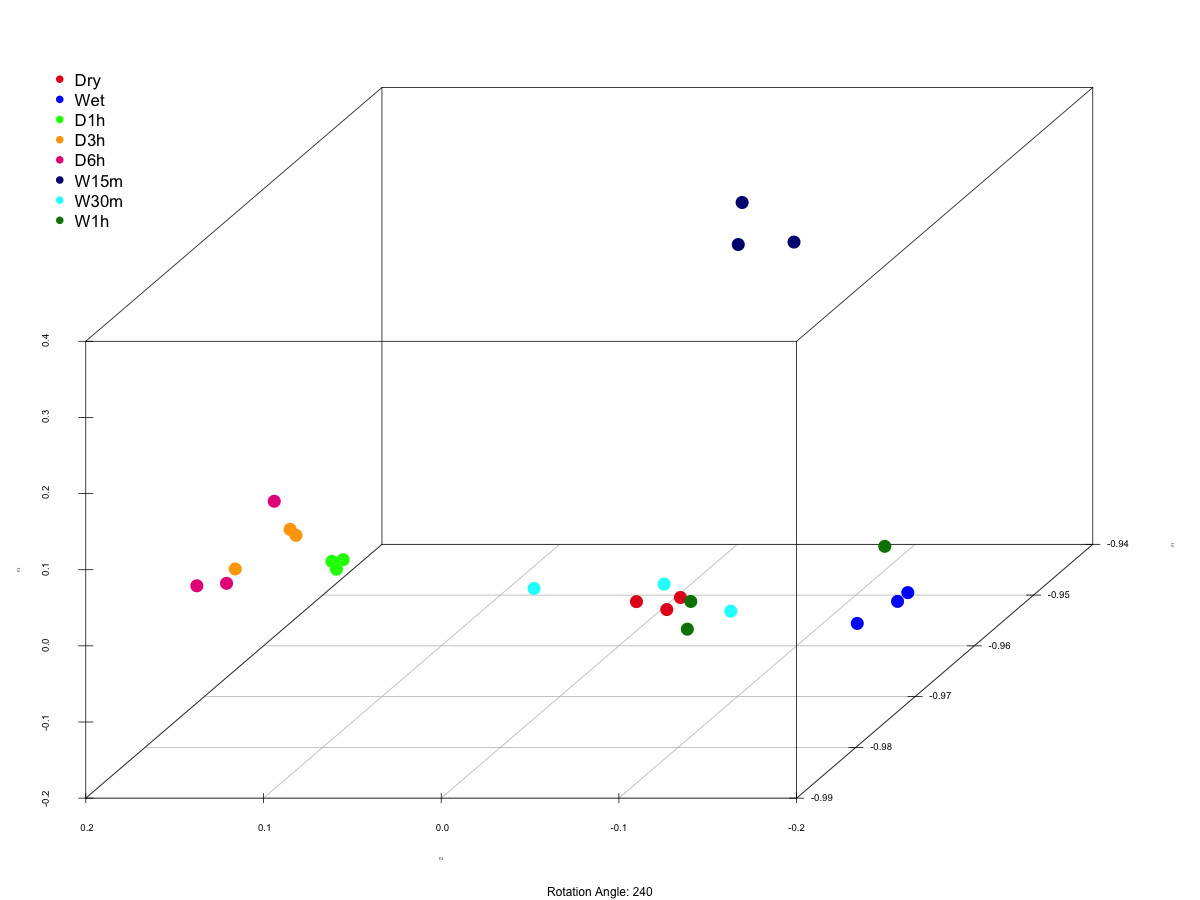

Supplement: Additional file 1 — Figures used in assessment of data quality in compressed format. [file 1471-2164-14-870-S1.zip › DataQualityControl/pca_all_norm.png]

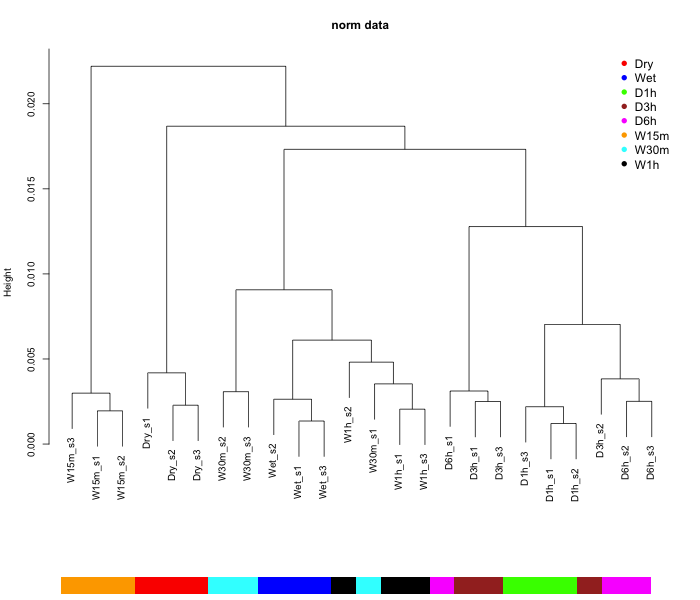

Supplement: Additional file 1 — Figures used in assessment of data quality in compressed format. [file 1471-2164-14-870-S1.zip › DataQualityControl/pearsonsClusteringAllSamples.png]

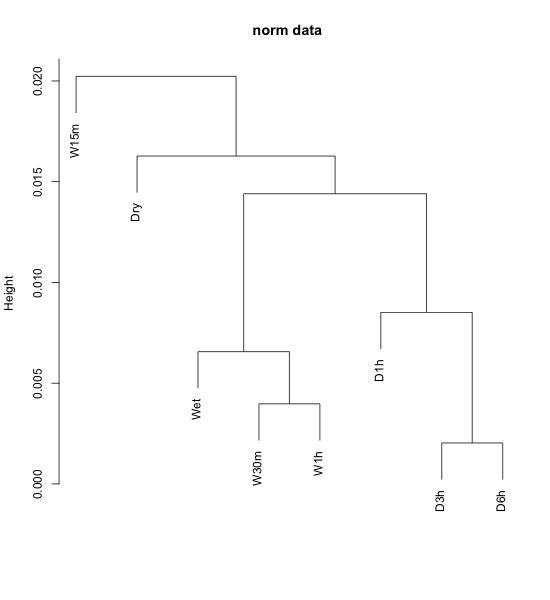

Supplement: Additional file 1 — Figures used in assessment of data quality in compressed format. [file 1471-2164-14-870-S1.zip › DataQualityControl/pearsonsClusteringGroups.png]

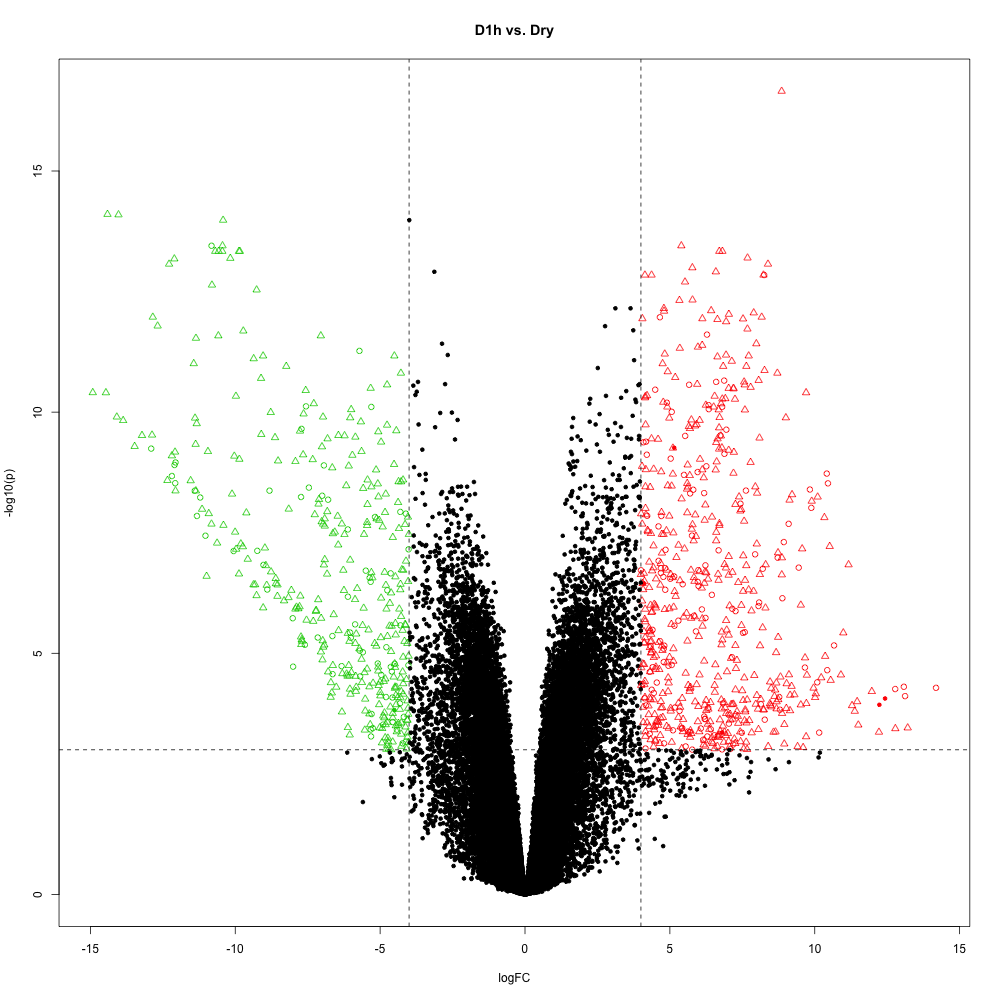

Supplement: Additional file 16 — Volcano plots for each comparison in compressed format. [file 1471-2164-14-870-S16.zip › volcanoPlots/Volcano_D1h_vs_Dry.png]

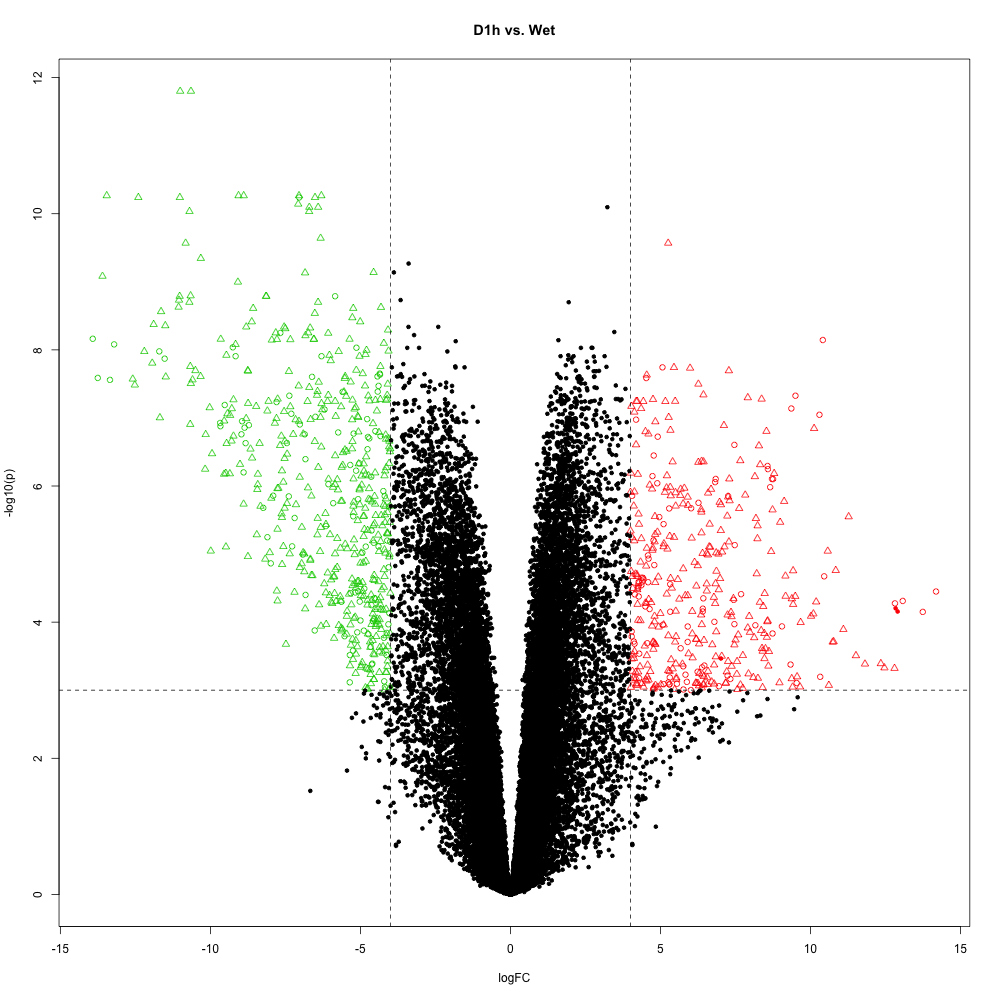

Supplement: Additional file 16 — Volcano plots for each comparison in compressed format. [file 1471-2164-14-870-S16.zip › volcanoPlots/Volcano_D1h_vs_Wet.png]

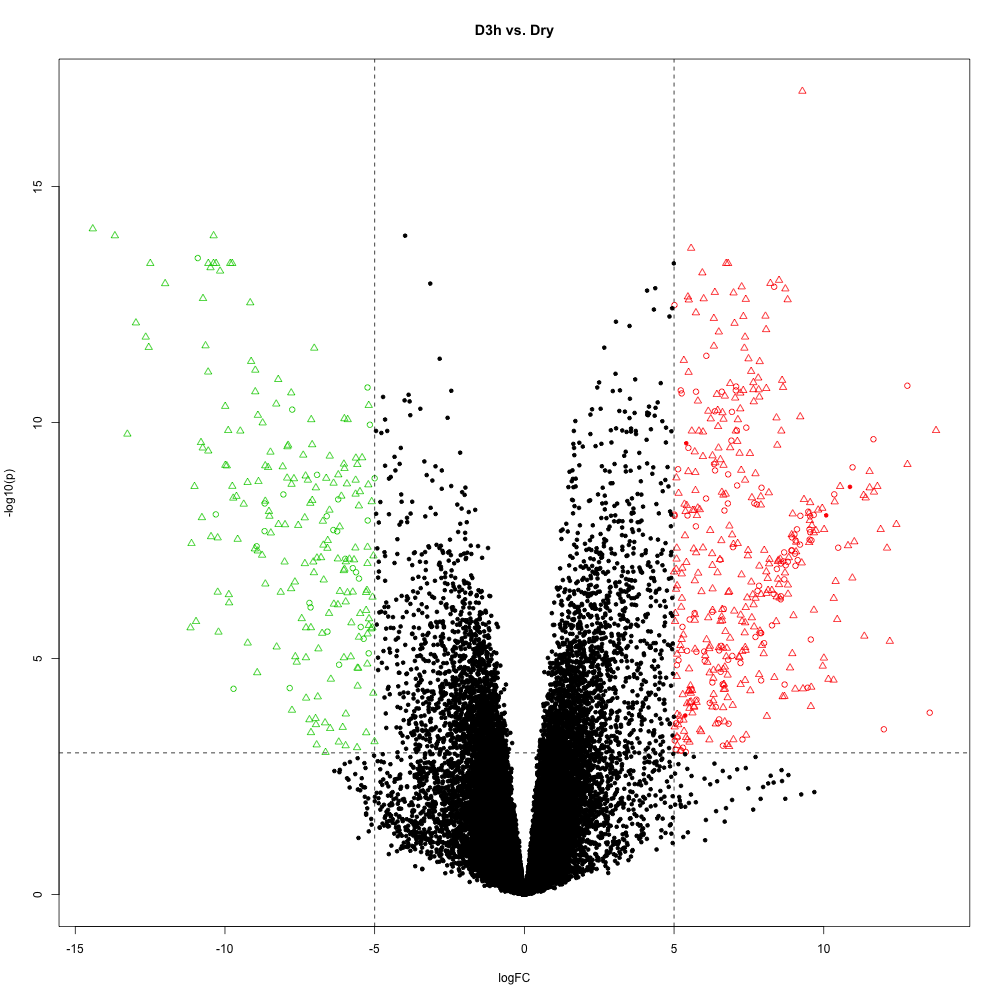

Supplement: Additional file 16 — Volcano plots for each comparison in compressed format. [file 1471-2164-14-870-S16.zip › volcanoPlots/Volcano_D3h_vs_Dry.png]

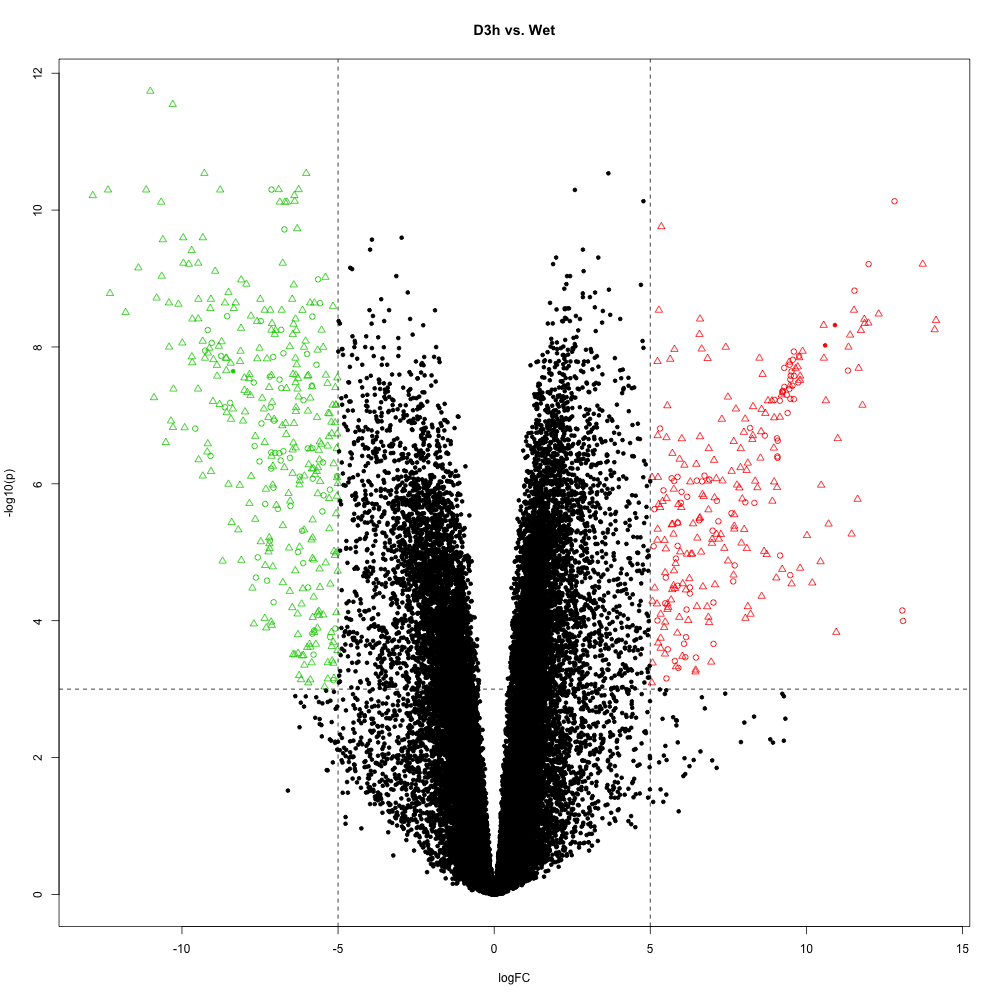

Supplement: Additional file 16 — Volcano plots for each comparison in compressed format. [file 1471-2164-14-870-S16.zip › volcanoPlots/Volcano_D3h_vs_Wet.png]

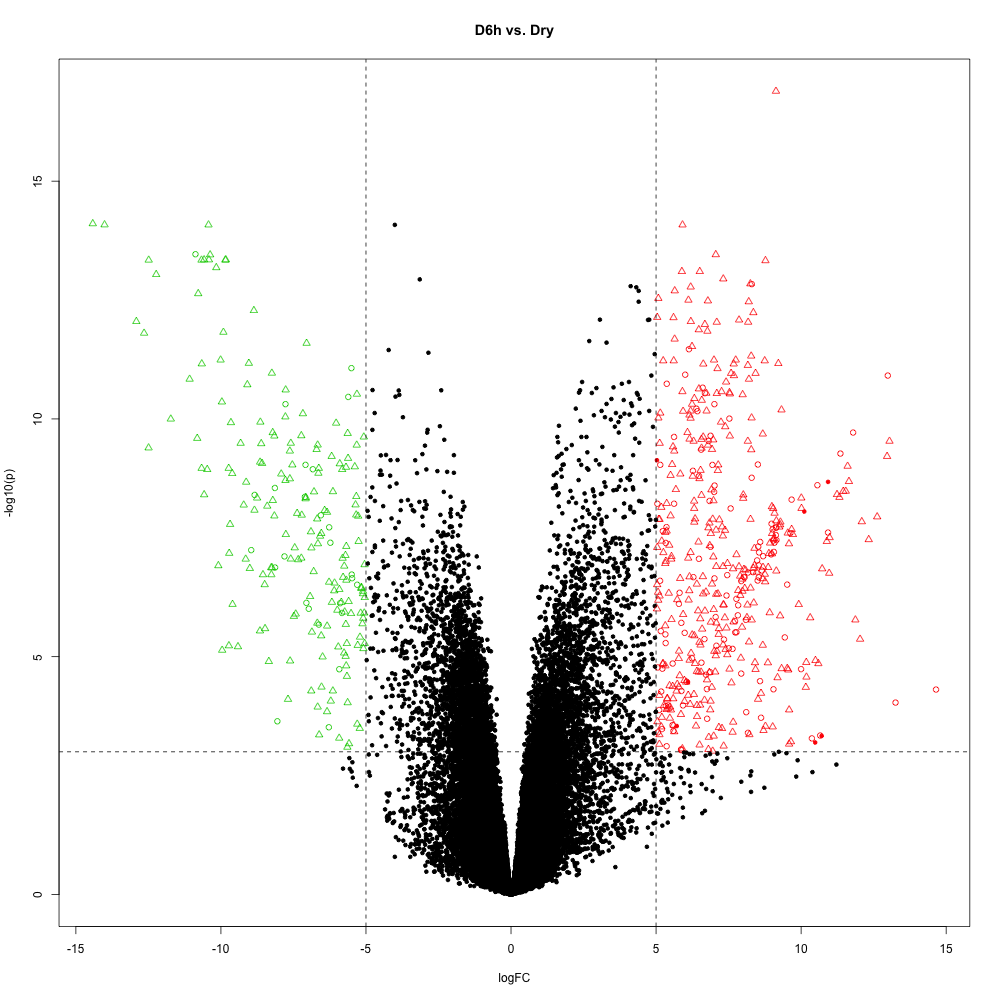

Supplement: Additional file 16 — Volcano plots for each comparison in compressed format. [file 1471-2164-14-870-S16.zip › volcanoPlots/Volcano_D6h_vs_Dry.png]

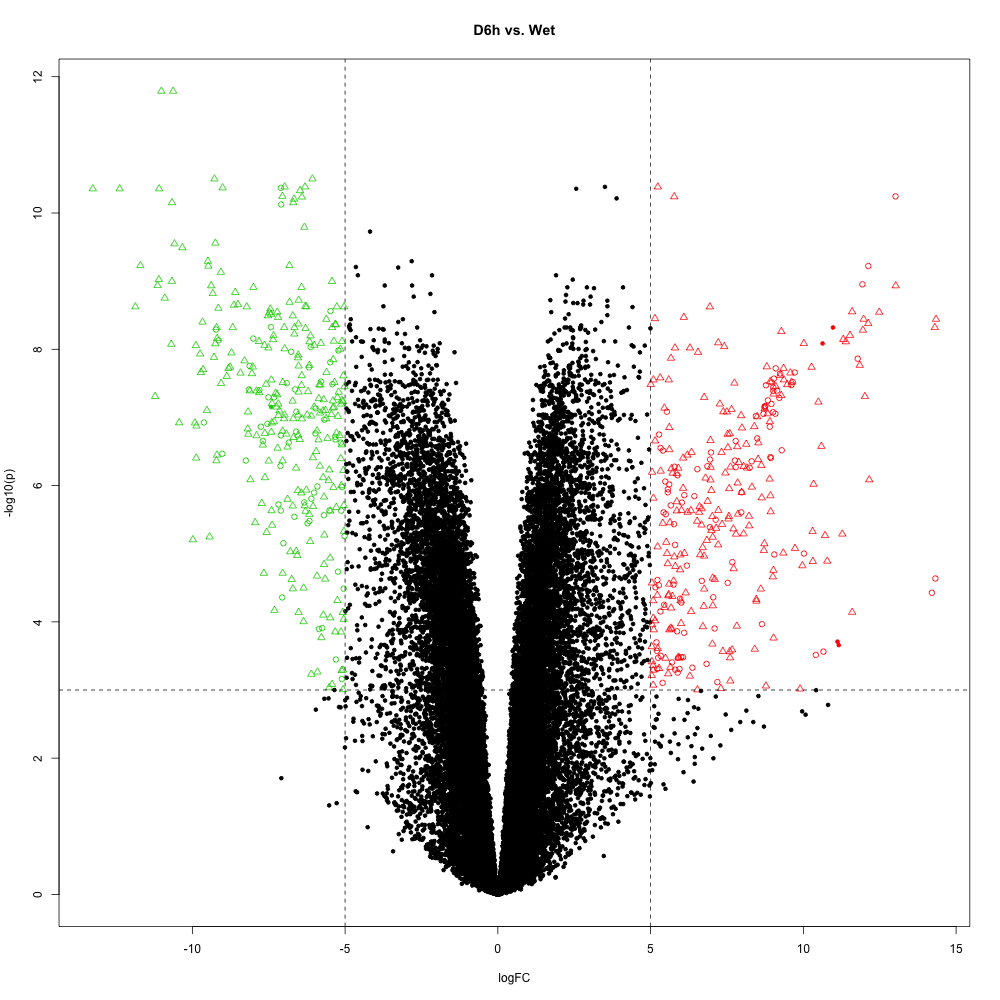

Supplement: Additional file 16 — Volcano plots for each comparison in compressed format. [file 1471-2164-14-870-S16.zip › volcanoPlots/Volcano_D6h_vs_Wet.png]

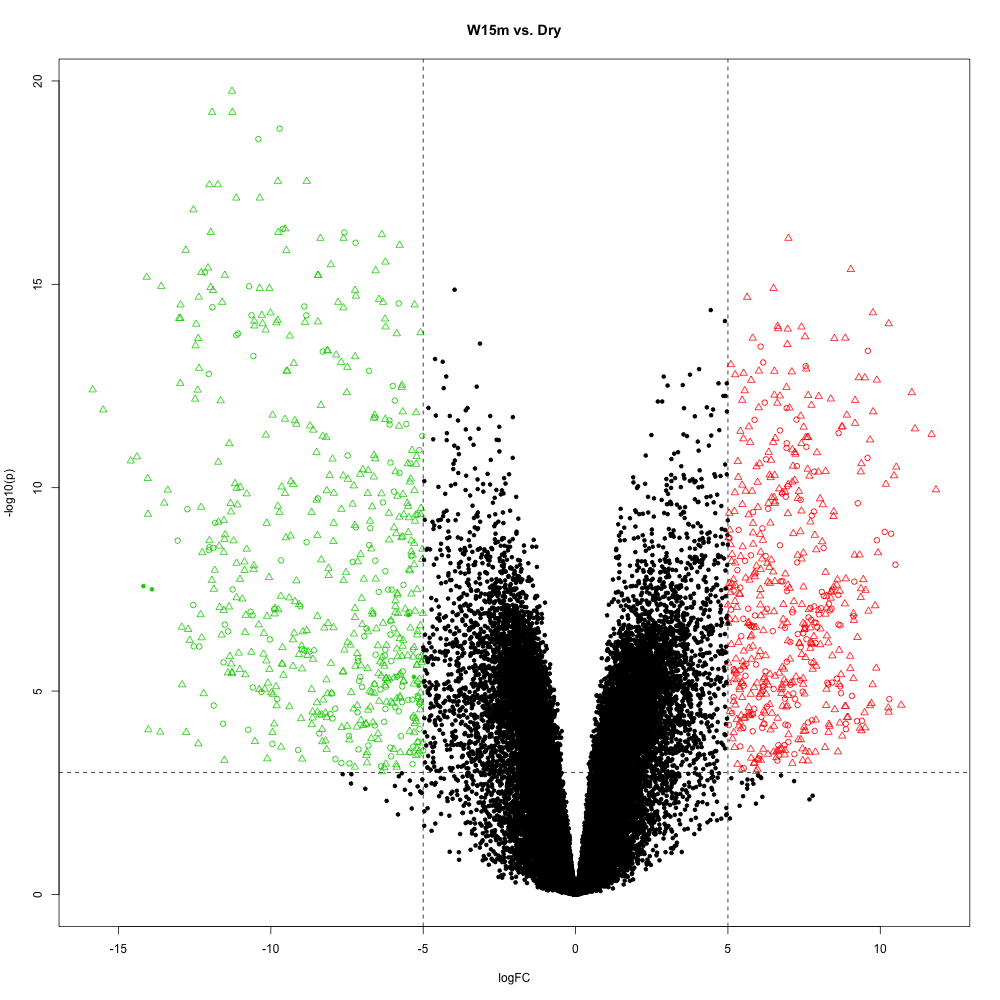

Supplement: Additional file 16 — Volcano plots for each comparison in compressed format. [file 1471-2164-14-870-S16.zip › volcanoPlots/Volcano_W15m_vs_Dry.png]

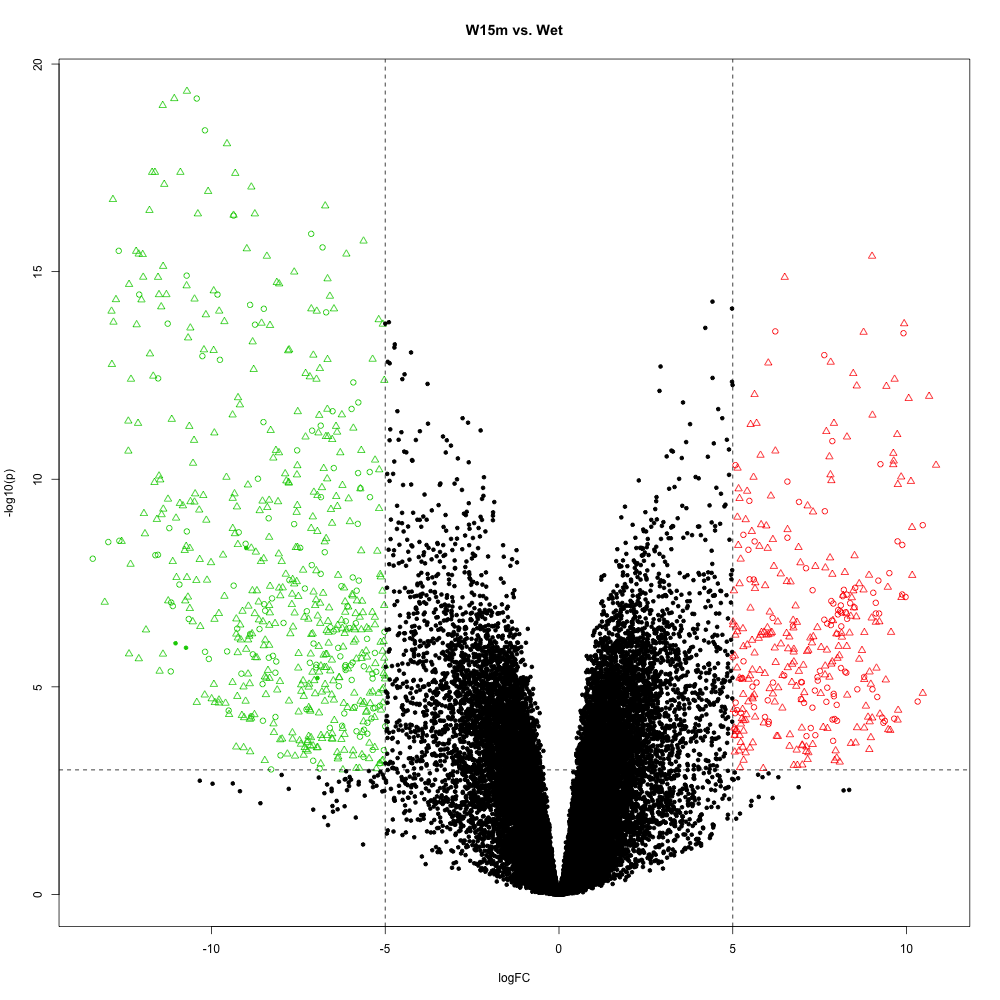

Supplement: Additional file 16 — Volcano plots for each comparison in compressed format. [file 1471-2164-14-870-S16.zip › volcanoPlots/Volcano_W15m_vs_Wet.png]

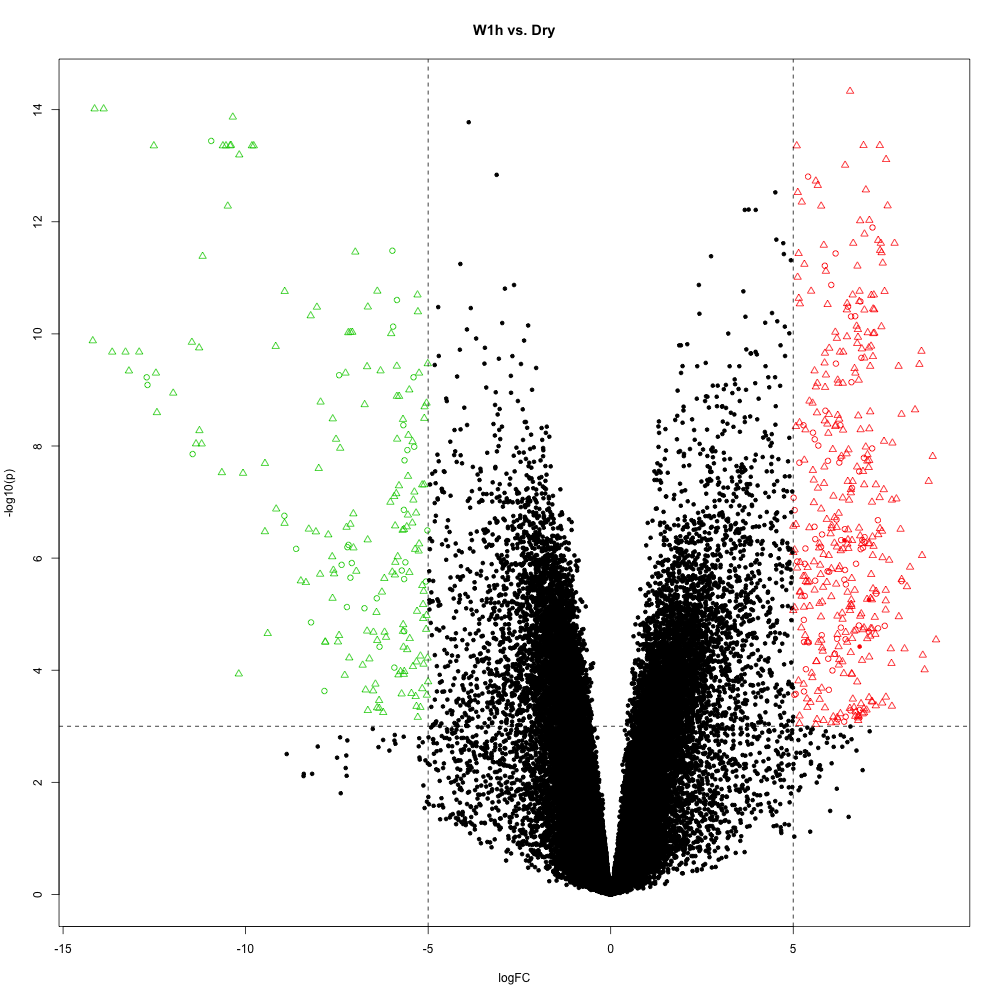

Supplement: Additional file 16 — Volcano plots for each comparison in compressed format. [file 1471-2164-14-870-S16.zip › volcanoPlots/Volcano_W1h_vs_Dry.png]

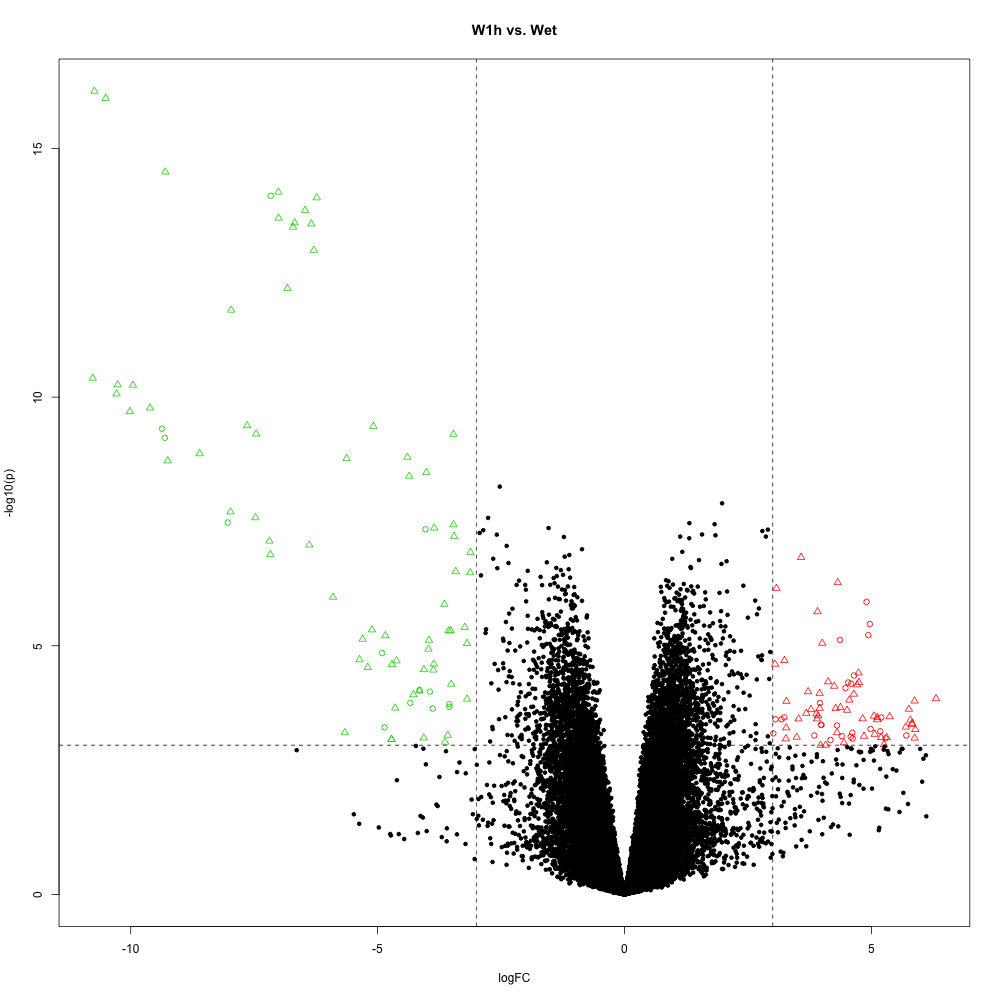

Supplement: Additional file 16 — Volcano plots for each comparison in compressed format. [file 1471-2164-14-870-S16.zip › volcanoPlots/Volcano_W1h_vs_Wet.png]

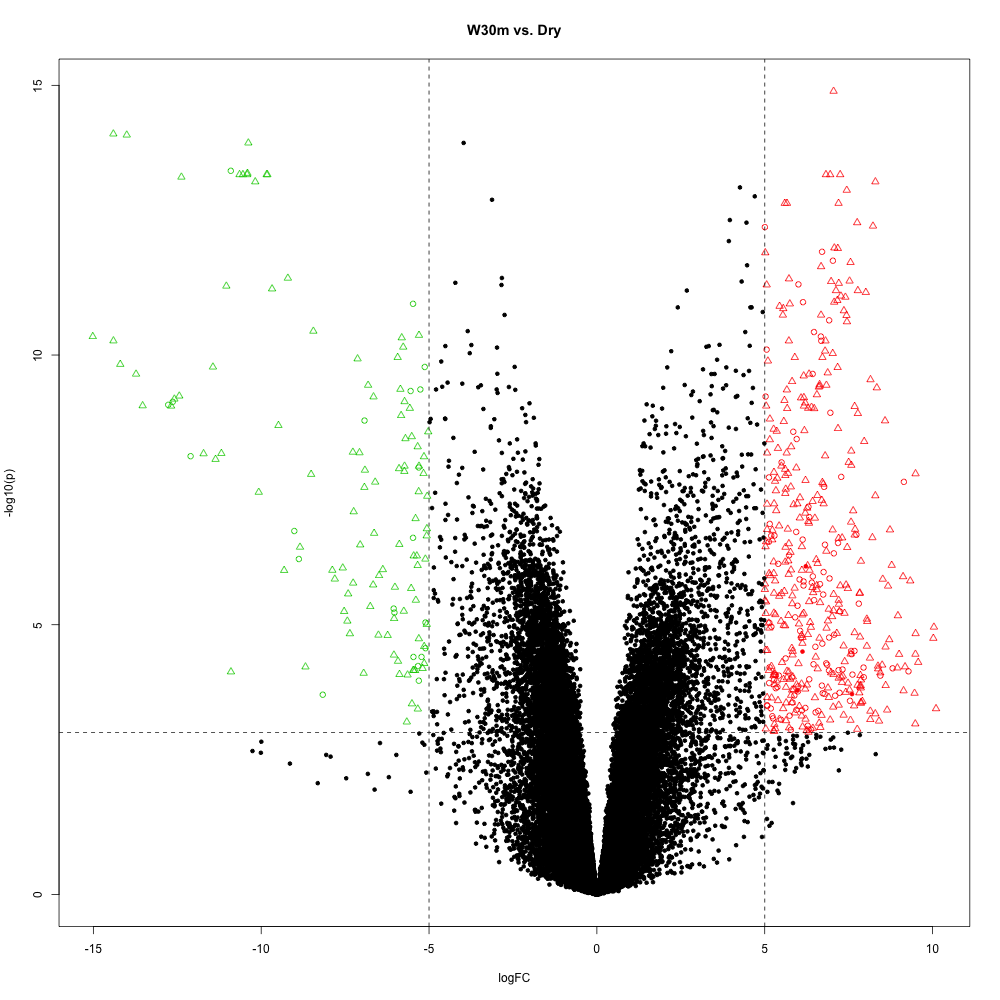

Supplement: Additional file 16 — Volcano plots for each comparison in compressed format. [file 1471-2164-14-870-S16.zip › volcanoPlots/Volcano_W30m_vs_Dry.png]

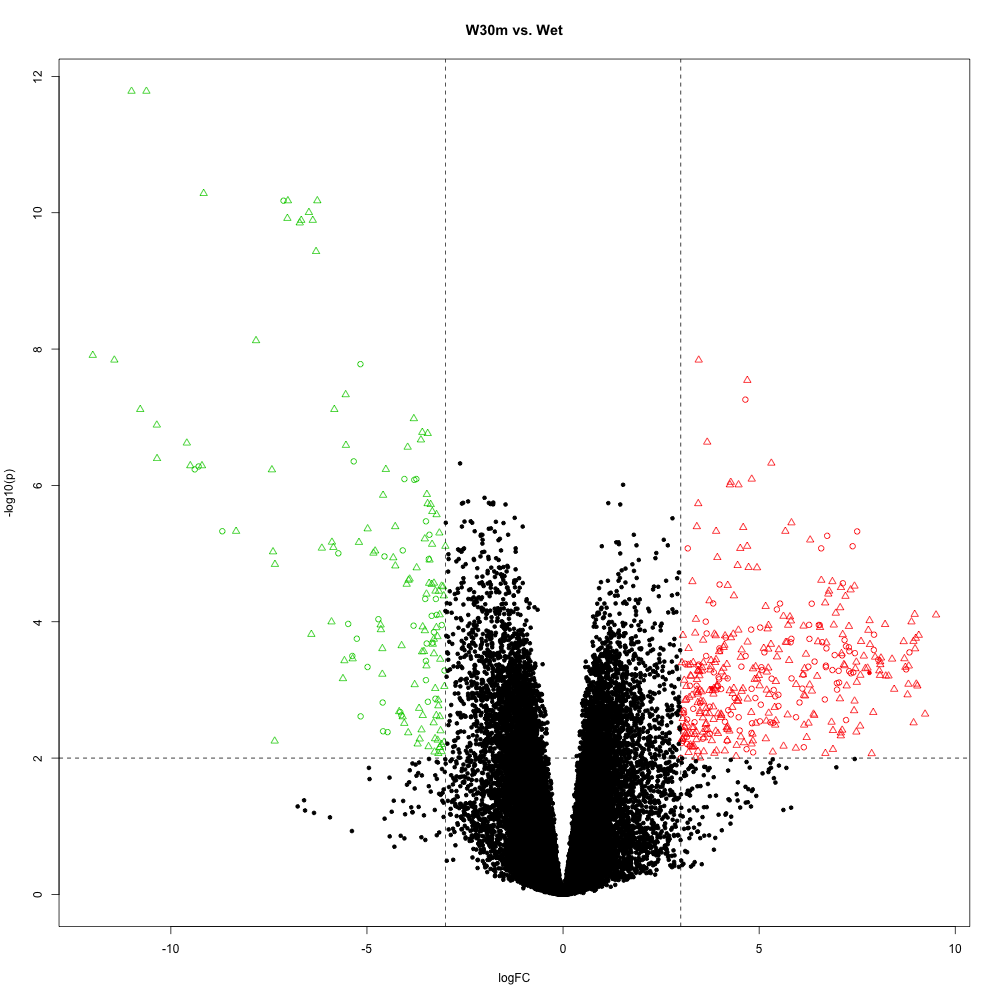

Supplement: Additional file 16 — Volcano plots for each comparison in compressed format. [file 1471-2164-14-870-S16.zip › volcanoPlots/Volcano_W30m_vs_Wet.png]

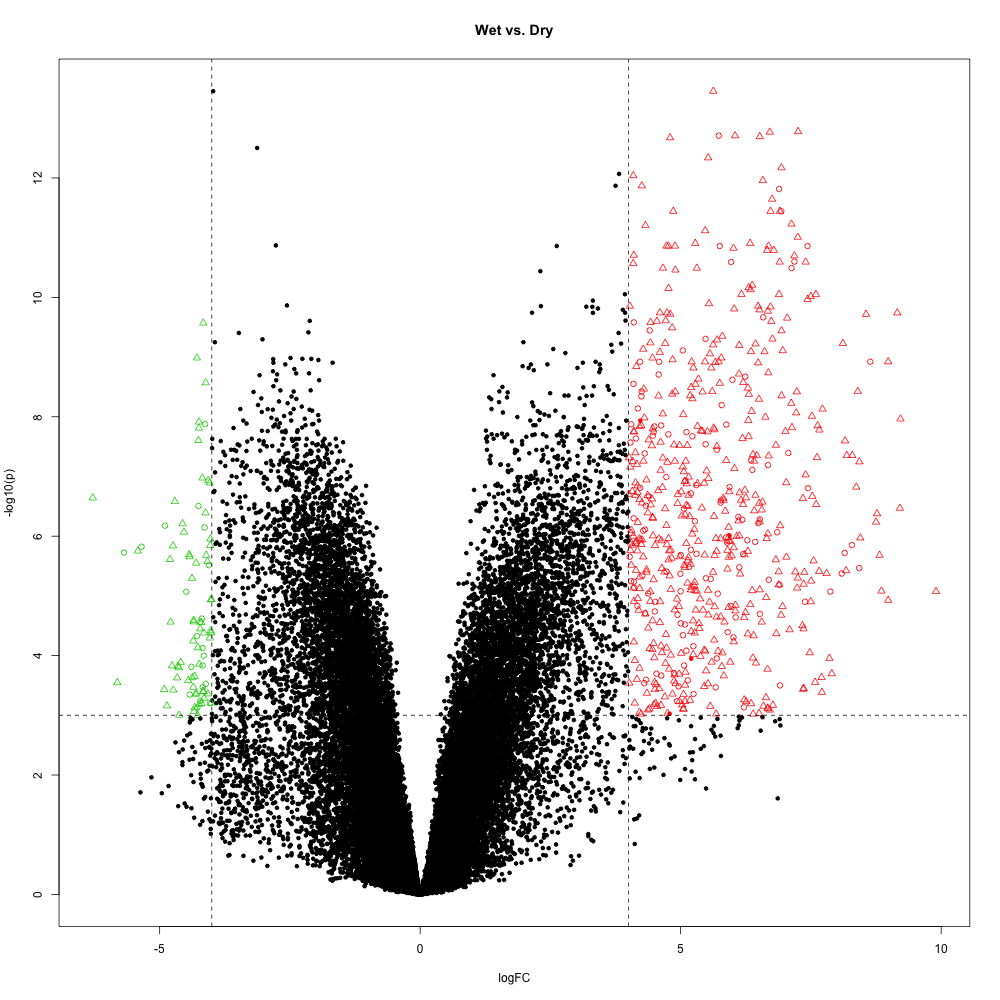

Supplement: Additional file 16 — Volcano plots for each comparison in compressed format. [file 1471-2164-14-870-S16.zip › volcanoPlots/Volcano_Wet_vs_Dry.png]

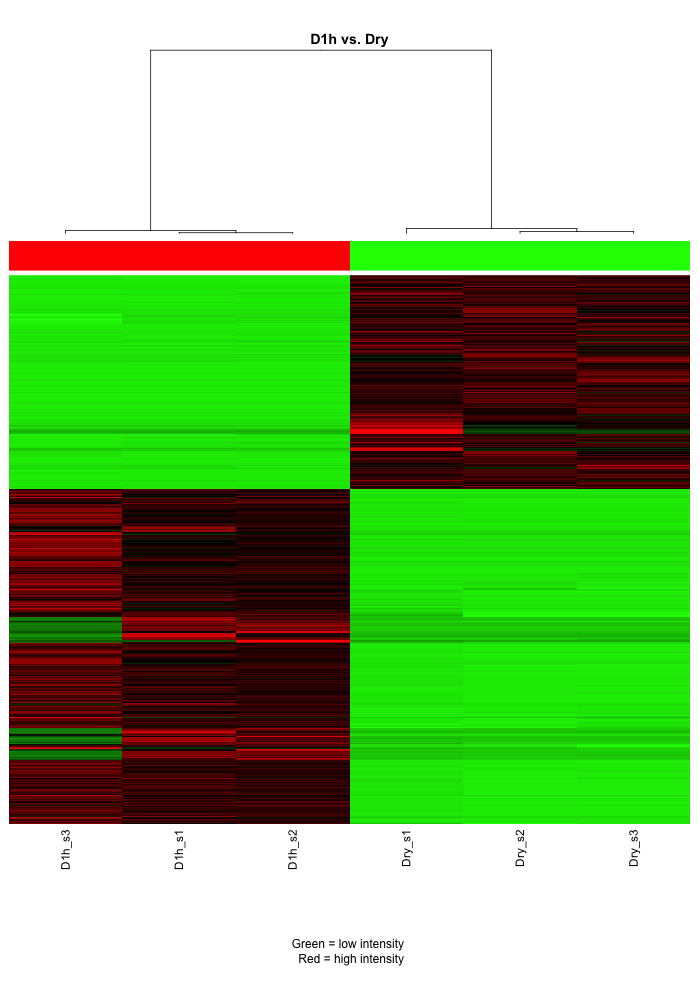

Supplement: Additional file 17 — Heat maps for each comparison in compressed format. [file 1471-2164-14-870-S17.zip › heatMaps/heatmap_D1h_VS_Dry.png]

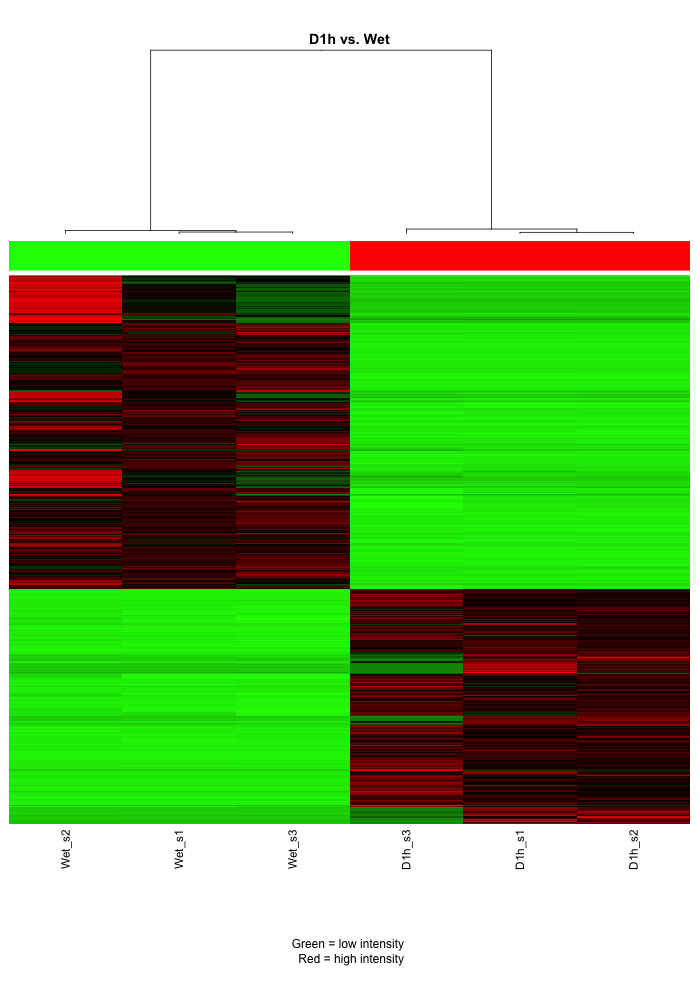

Supplement: Additional file 17 — Heat maps for each comparison in compressed format. [file 1471-2164-14-870-S17.zip › heatMaps/heatmap_D1h_VS_Wet.png]

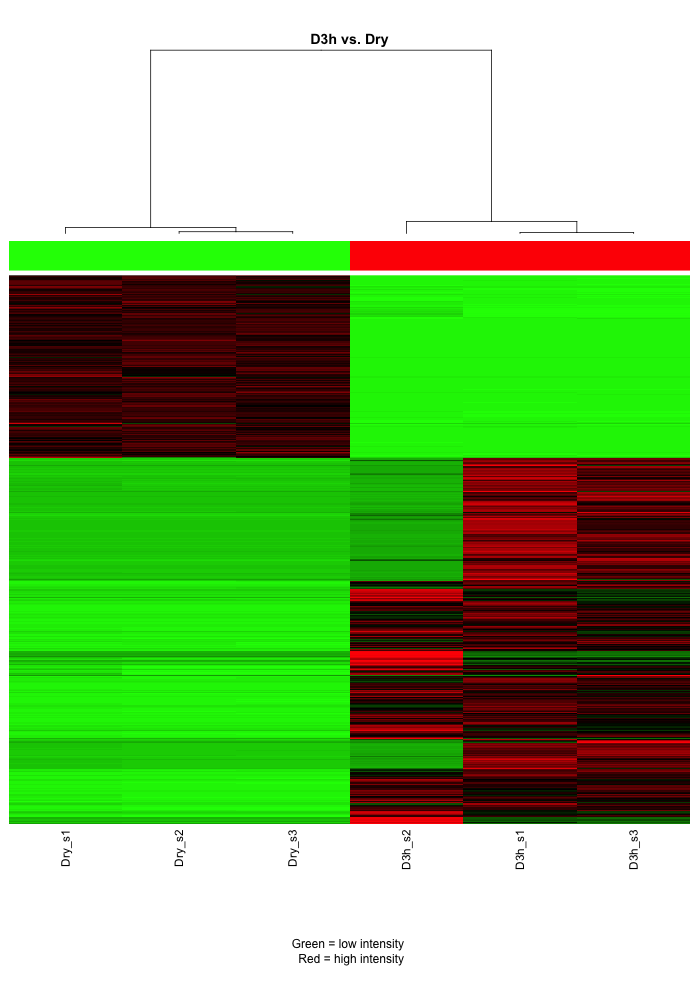

Supplement: Additional file 17 — Heat maps for each comparison in compressed format. [file 1471-2164-14-870-S17.zip › heatMaps/heatmap_D3h_VS_Dry.png]

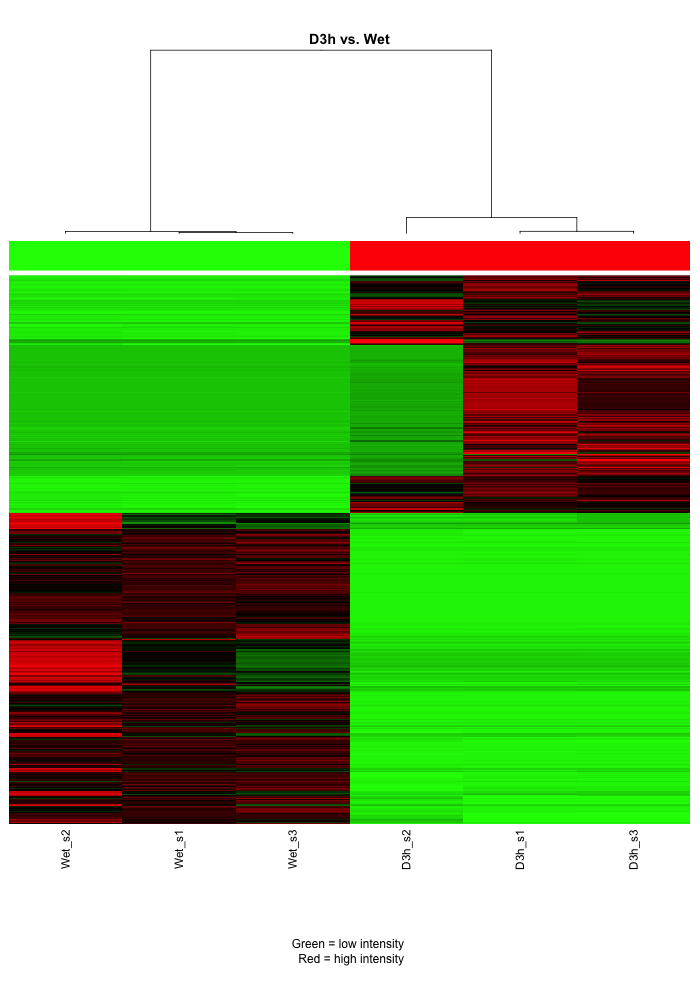

Supplement: Additional file 17 — Heat maps for each comparison in compressed format. [file 1471-2164-14-870-S17.zip › heatMaps/heatmap_D3h_VS_Wet.png]

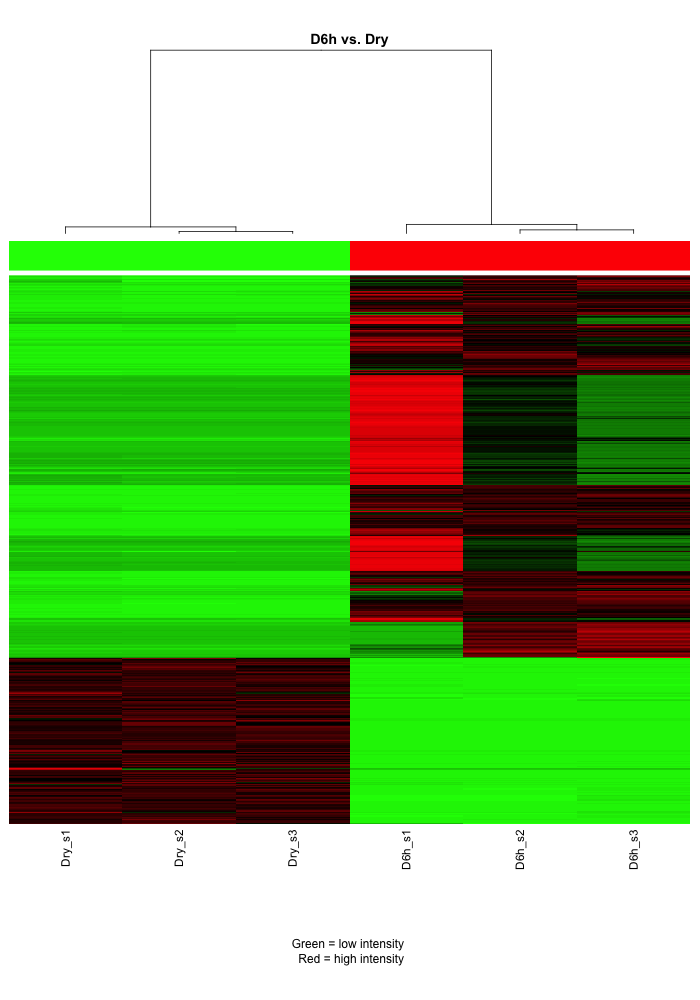

Supplement: Additional file 17 — Heat maps for each comparison in compressed format. [file 1471-2164-14-870-S17.zip › heatMaps/heatmap_D6h_VS_Dry.png]

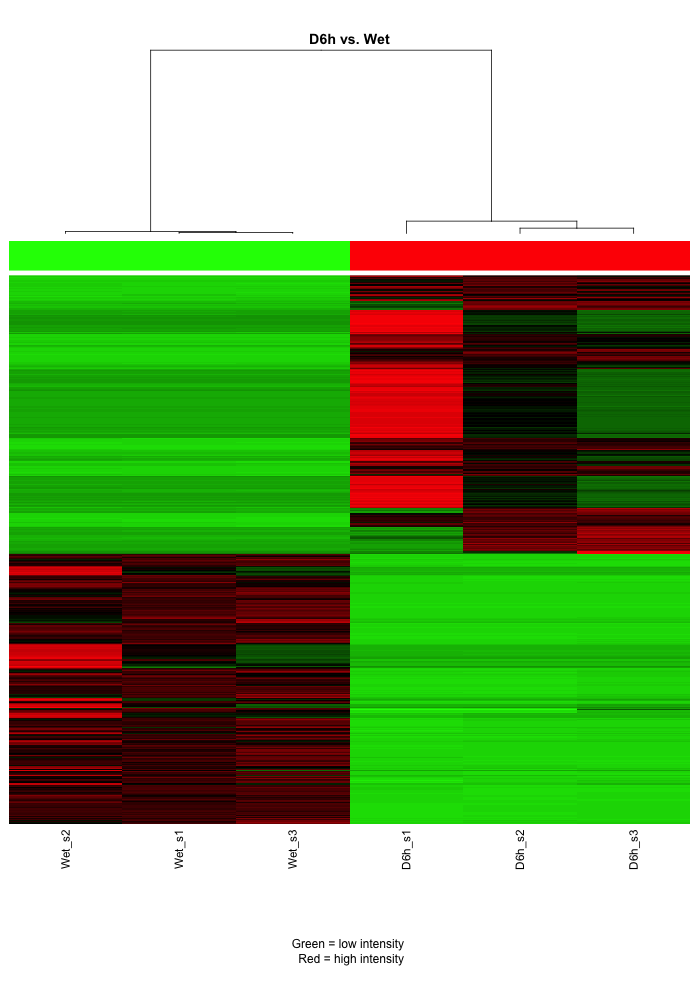

Supplement: Additional file 17 — Heat maps for each comparison in compressed format. [file 1471-2164-14-870-S17.zip › heatMaps/heatmap_D6h_VS_Wet.png]

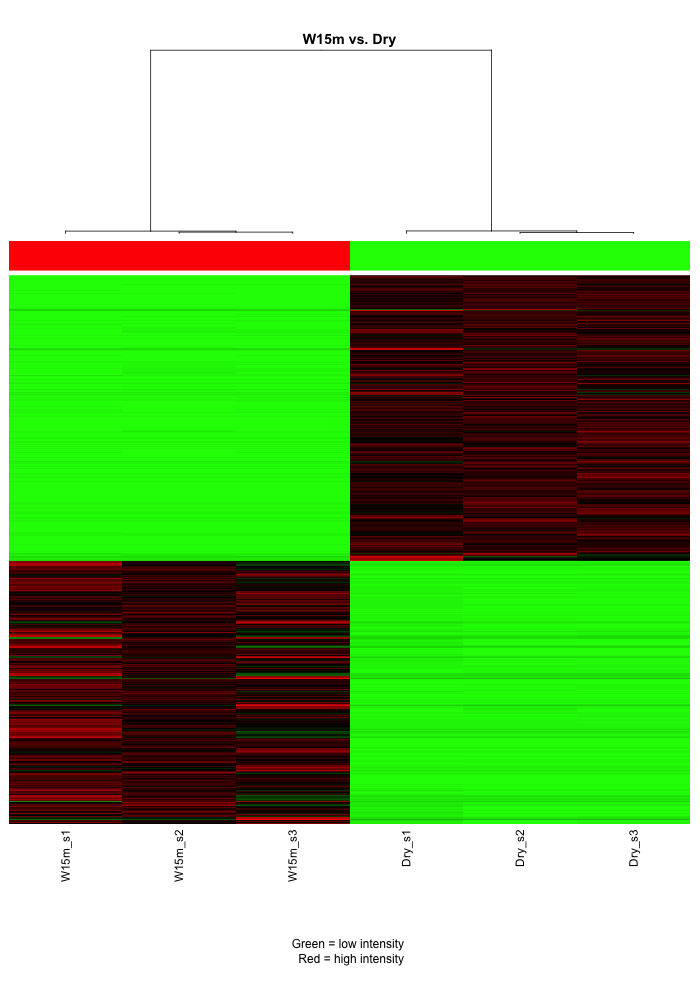

Supplement: Additional file 17 — Heat maps for each comparison in compressed format. [file 1471-2164-14-870-S17.zip › heatMaps/heatmap_W15m_VS_Dry.png]

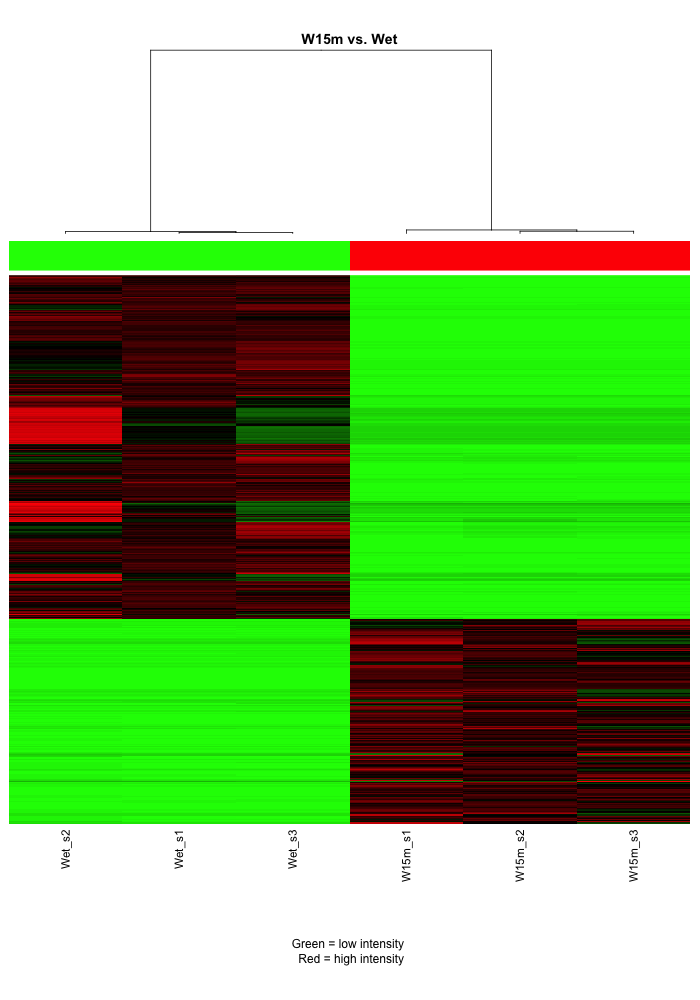

Supplement: Additional file 17 — Heat maps for each comparison in compressed format. [file 1471-2164-14-870-S17.zip › heatMaps/heatmap_W15m_VS_Wet.png]

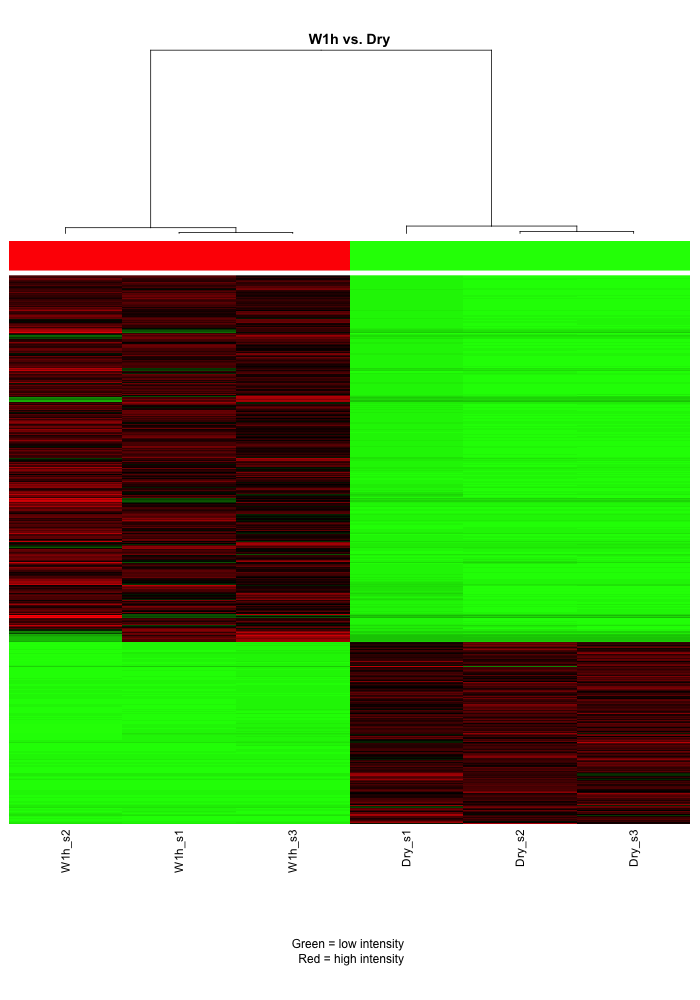

Supplement: Additional file 17 — Heat maps for each comparison in compressed format. [file 1471-2164-14-870-S17.zip › heatMaps/heatmap_W1h_VS_Dry.png]

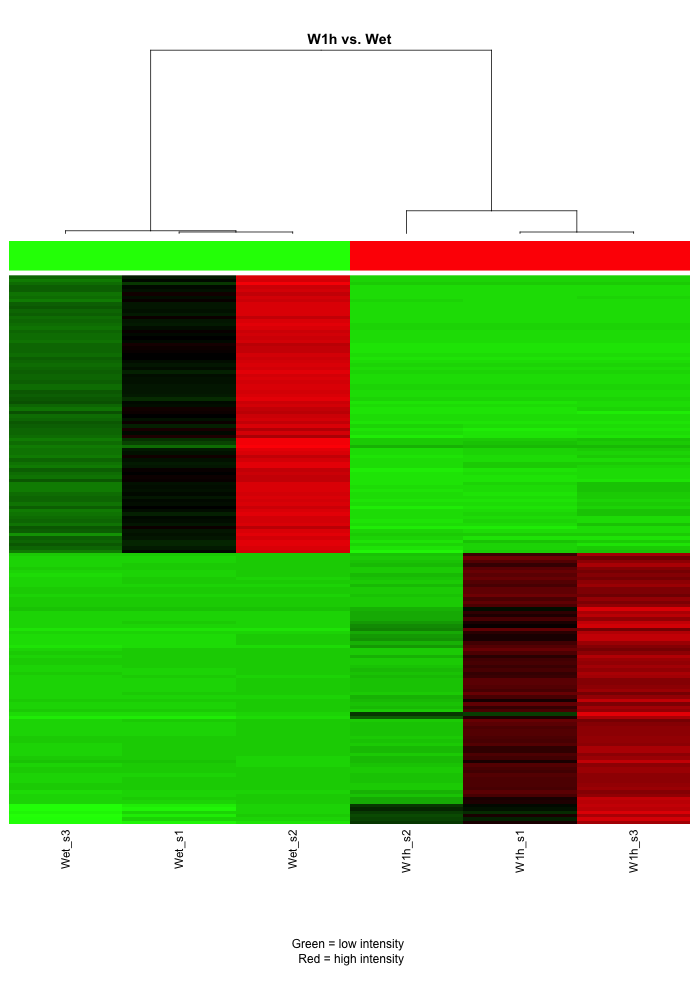

Supplement: Additional file 17 — Heat maps for each comparison in compressed format. [file 1471-2164-14-870-S17.zip › heatMaps/heatmap_W1h_VS_Wet.png]

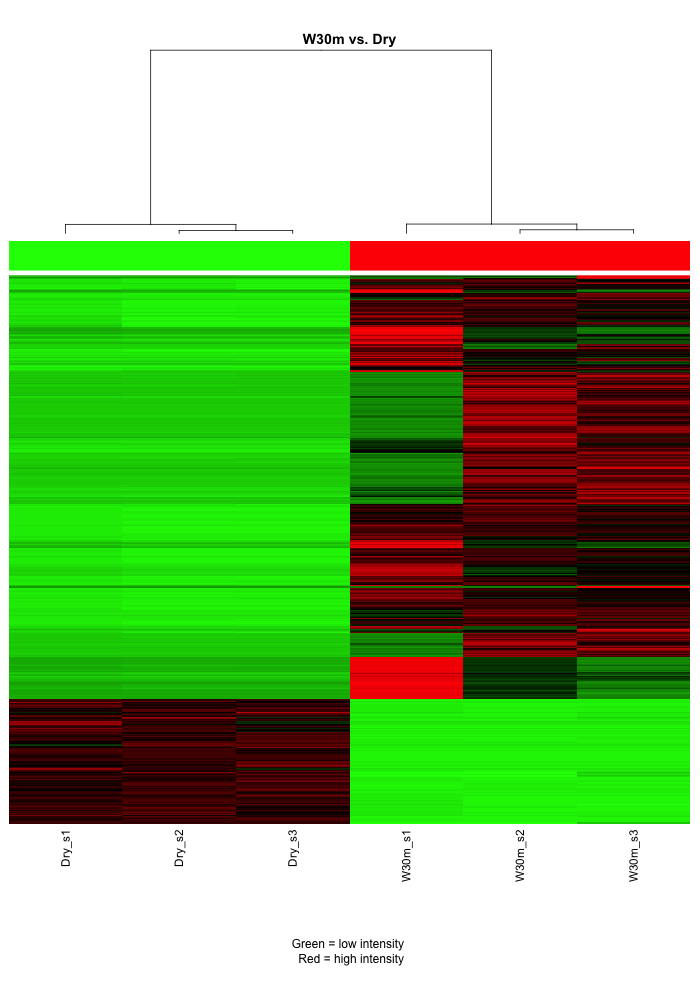

Supplement: Additional file 17 — Heat maps for each comparison in compressed format. [file 1471-2164-14-870-S17.zip › heatMaps/heatmap_W30m_VS_Dry.png]

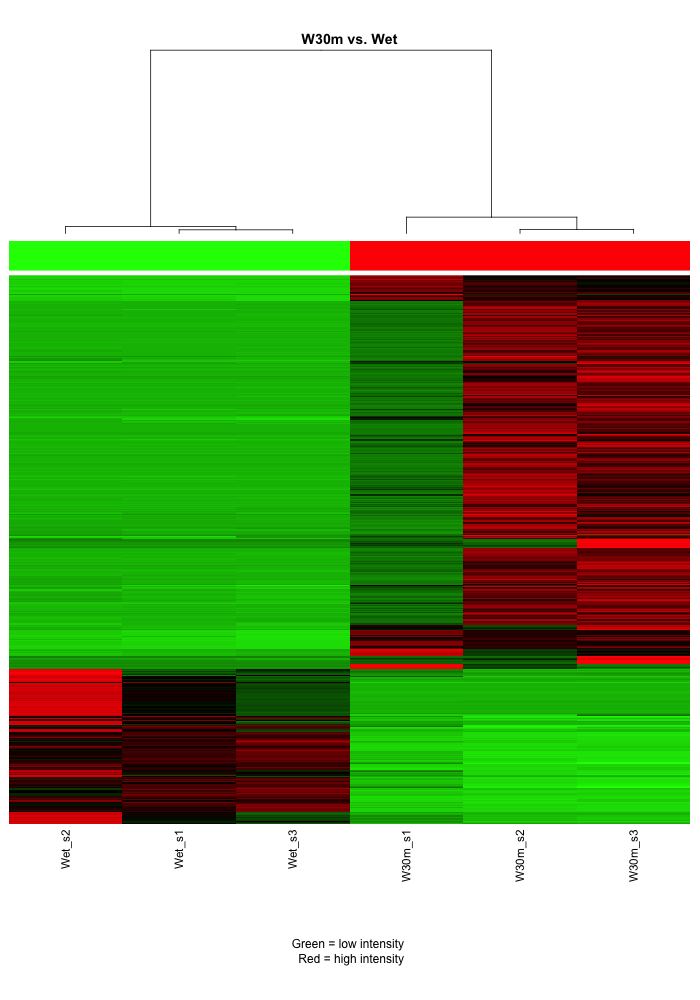

Supplement: Additional file 17 — Heat maps for each comparison in compressed format. [file 1471-2164-14-870-S17.zip › heatMaps/heatmap_W30m_VS_Wet.png]

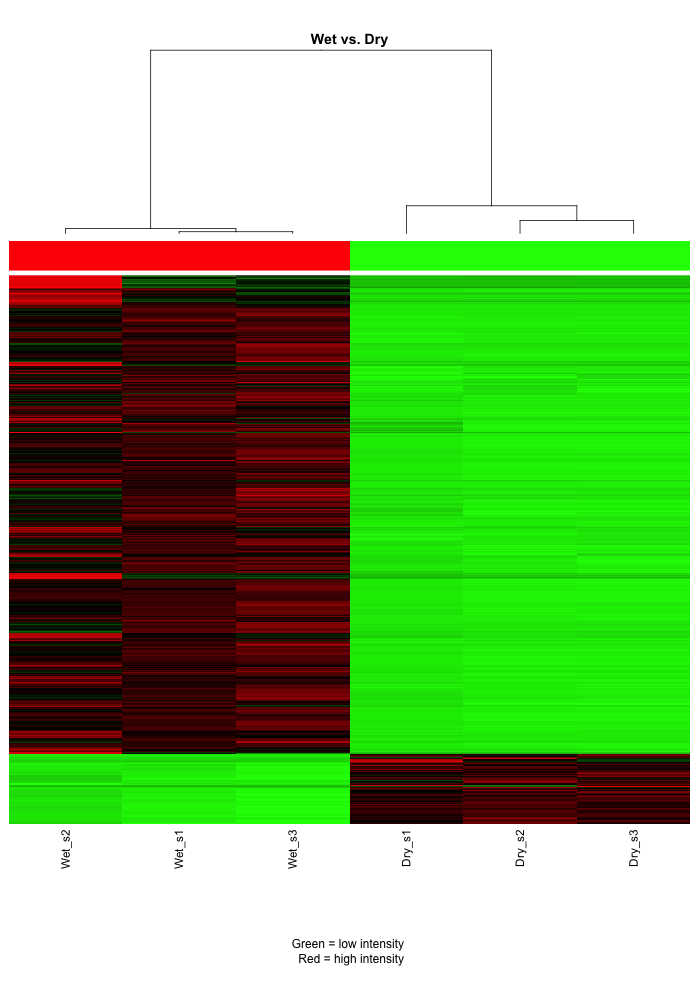

Supplement: Additional file 17 — Heat maps for each comparison in compressed format. [file 1471-2164-14-870-S17.zip › heatMaps/heatmap_Wet_VS_Dry.png]

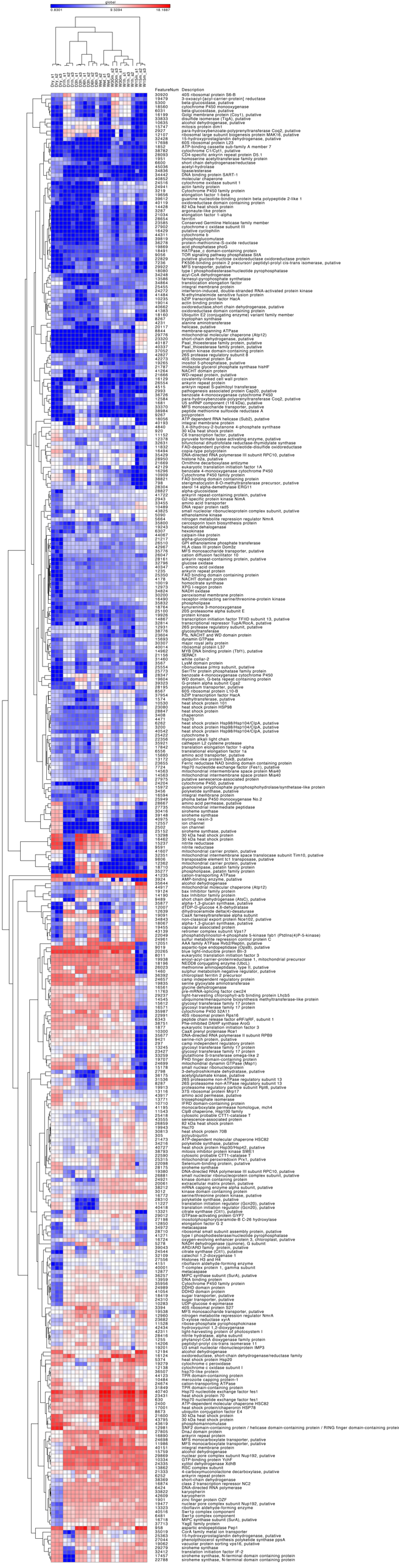

Supplement: Additional file 19 — Clustering heat map of the DE genes. [file 1471-2164-14-870-S19.pdf]
