# Supplementary material for: Whole transcriptome characterization of the effects of dehydration and rehydration on Cladonia rangiferina, the grey reindeer lichen
Source: BMC Genomics. 2013 Dec 10;14:870. doi: 10.1186/1471-2164-14-870 (PMC3878897; doi:10.1186/1471-2164-14-870)
Supplement: Additional file 18 — The amount common differentially expressed transcripts in the comparisons. [file 1471-2164-14-870-S18.docx]

**The number of common differentially expressed transcripts in the comparisons**

The numbers are given in the form: ‘no1 (no2)’, where no1 gives the total of overlapping differentially expressed array transcriptss between the comparisons, and no2 gives the number of disagreeing DE array transcripts (up or down in first comparison and vice versa in the other).

| Comparison | Wet vs. Dry | W15m vs. Dry | W30 vs. Dry | W1h vs. Dry | W15m vs. Wet | W30m vs. Wet | W1h vs. Wet | D1h vs. Dry | D3h vs. Dry | D6h vs. Dry | D1h vs. Wet | D3h vs. Wet |
| --- | --- | --- | --- | --- | --- | --- | --- | --- | --- | --- | --- | --- |
| W15m vs. Dry | 233 (10) |  |  |  |  |  |  |  |  |  |  |  |
| W30m vs. Dry | 305 (0) | 329 (5) |  |  |  |  |  |  |  |  |  |  |
| W1h vs. Dry | 400 (0) | 311 (9) | 410 (0) |  |  |  |  |  |  |  |  |  |
| W15m vs. Wet | 165 (158) | 609 (0) | 189 (81) | 181 (128) |  |  |  |  |  |  |  |  |
| W30m vs. Wet | 60 (49) | 200 (4) | 206 (0) | 83 (8) | 235 (3) |  |  |  |  |  |  |  |
| W1h vs. Wet | 16 (2) | 105 (0) | 137 (0) | 88 (0) | 95 (0) | 136 (0) |  |  |  |  |  |  |
| D1h vs. Dry | 293 (16) | 407 (10) | 416 (6) | 377 (13) | 266 (72) | 216 (4) | 124 (0) |  |  |  |  |  |
| D3h vs. Dry | 212 (2) | 325 (7) | 288 (1) | 271 (1) | 203 (55) | 129 (3) | 70 (0) | 423 (3) |  |  |  |  |
| D6h vs. Dry | 215 (2) | 336 (6) | 283 (1) | 270 (1) | 205 (53) | 133 (2) | 71 (0) | 464 (3) | 597 (0) |  |  |  |
| D1h vs.  Wet | 186 (171) | 239 (48) | 200 (42) | 171 (91) | 291 (9) | 325 (1) | 125 (0) | 456 (0) | 218 (14) | 235 (14) |  |  |
| D3h vs.  Wet | 151 (146) | 186 (33) | 130 (56) | 132 (94) | 263 (3) | 197 (3) | 65 (1) | 218 (16) | 330 (0) | 315 (0) | 359 (2) |  |
| D6h vs.  Wet | 140 (135) | 187 (32) | 128 (55) | 125 (90) | 263 (3) | 212 (1) | 65 (1) | 240 (15) | 312 (0) | 329 (0) | 391 (2) | 534 (0) |
