# Supplementary material for: Whole transcriptome characterization of the effects of dehydration and rehydration on Cladonia rangiferina, the grey reindeer lichen
Source: BMC Genomics. 2013 Dec 10;14:870. doi: 10.1186/1471-2164-14-870 (PMC3878897; doi:10.1186/1471-2164-14-870)
Supplement: Additional file 21 — qRT-PCR primer sequences. [file 1471-2164-14-870-S21.docx]

| Primer | Sequence |
| --- | --- |
| Cr_lrc491_left | cacattgggactgagacacg |
| Cr_lrc491_right | cgctcattgtccaaaattcc |
| Cr_DMini_2305E01_left | caaccgagacaccacacaat |
| Cr_DMini_2305E01_right | tggaccgtaatcgacatacatc |
| Cr_lrc323_left | gctgccgacagaggatactg |
| Cr_lrc323_right | tcggattgatacttcgtctgg |
| Cr_c10766_left | tggcttcacagagaaggacttt |
| Cr_c10766_right | atcttgcaacccctgtcaaa |
| Cr_c3825_left | tggctatcatatacggaaccttg |
| Cr_c3825_right | tttgctggaagacgataggg |
| Cr_c4168_left | ggcgaggcttgaaagcta |
| Cr_c4168_right | ggtatgctccatatgtgattgc |
| Cr_lrc5_left | ttaggttggcggagaaaagtt |
| Cr_lrc5_right | gaccatcttaaacgaatgacataca |
| Cr_c15269_left | ggaagtataagggttcccagtca |
| Cr_c15269_right | aacccgaatatgctatgacacttag |
| Cr_DMini_673H04_left | ccgagctgtccaagctgt |
| Cr_DMini_673H04_right | aaacgtactgaaattgcgactct |
| Cr_c18326_left | tcgtaatacactctgacggatctc |
| Cr_c18326_right | ctctcgggtggtgttctagg |
| Cr_lrc282_left | accccagcatcaacattctc |
| Cr_lrc282_right | aagggttgttcgttgtttgct |

Supplementary Table 1. Primers used in the qPCR validation.
